# Bayes Factors show evidence against systematic relationships between the anchoring effect and the Big Five personality traits

**DOI:** 10.1038/s41598-021-86429-2

**Published:** 2021-03-29

**Authors:** Sebastian Schindler, Jan Querengässer, Maximilian Bruchmann, Nele Johanna Bögemann, Robert Moeck, Thomas Straube

**Affiliations:** 1 Institute of Medical Psychology and Systems Neuroscience, University of Muenster, Von-Esmarch-Str. 52, 48149 Muenster, Germany; 2 Otto Creutzfeldt Center for Cognitive and Behavioral Neuroscience, University of Muenster, Muenster, Germany; 3 Department of Psychology, University of Konstanz, Constance, Germany

**Keywords:** Human behaviour, Psychology

## Abstract

Examining personality traits as predictors of human behaviour is of high interest. There are several but inconclusive reported relationships of personality and the susceptibility to the “anchoring effect”, a tendency to adjust estimates towards a given anchor. To provide an answer to variably reported links between personality traits and the anchoring effect, we collected data from 1000 participants in the lab and validated typical anchoring effects and representative personality scores of the sample. Using Bayesian statistical data analyses, we found evidence for the absence of a relationship between anchoring effects and personality scores. We, therefore, conclude that there are no specific personality traits that relate to a higher susceptibility to the anchoring effect. The lack of a relationship between personality and the susceptibility to the anchoring effect might be due to the specific anchoring design, be limited to specific cognitive domains, or the susceptibility to anchors might reflect no reliable individual cognitive phenomena. In the next step, studies should examine the reliability of anchoring effects on the individual level, and testing relationships of individual traits and anchoring effects for other types of anchors, anchoring designs, or cognitive domains.

## Introduction

Research shows that a small number of stable personality traits characterizes individual differences between humans. These personality traits are relatively consistent, even when taking in a long-term perspective^1,2^. The Big Five Model, for instance, is one of the most accepted and well-established models to describe individual personality traits^3^. The broad applicability of personality as a predictor of human behavior has led to more than a hundred studies relating personality differences to cognitive measures^4–7^. A well-known examined cognitive bias is the anchoring effect. It describes the phenomenon that people confronted with a “guesstimation task” base their estimates on previously perceived numerical information^8^. Here, a review on influencing factors concluded that anchoring effects seem to be related to personality, but results are highly divergent and thus further studies are needed^9^.

In experimental studies presenting distinct numerical anchors to different groups, the anchoring effect becomes evident in robust between-group differences in estimates for high compared to low anchors^10–12^. Dating back to the first published findings on the anchoring effect, Tversky and Kahnemann^13^ already suggested that individual differences could be determinants of estimation behavior, i.e. some individuals might be more prone to anchoring effects. Subsequently, researchers have begun to relate anchoring effects to individual differences in personality^14–19^, However, the evidence is highly mixed: McElroy and Dowd^16^ reported that high levels of openness for experience are related to a higher susceptibility to the anchoring effect on estimating the Missipi river length. To this end, they used a median-split on openness, derived from the two-item version of the Big Five personality model (TIPI)^20^, and tested for between-group interaction effects. In contrast, Furnham and colleagues^18^ could not replicate this finding with a similar between-group analysis. They used the NEO-FFI inventory^3^ and observed for one of four anchor questions a relationship of lower levels of extraversion with larger anchoring effects^18^. Eroglu and Croxton^15^ relied on a different Big Five personality model^21^ and examined forecast biases in the work environment by using a regression model, observing a relationship of high levels of conscientiousness and agreeableness and low levels of extraversion with anchoring effects. Caputo^14^ used the TIPI^20^ inventory and an individual regression model of personality traits with deviations from the anchor information provided about the question when the Taj Mahal was completed. This showed larger anchoring effects for low levels of conscientiousness and low levels of openness. Welsh and colleagues^17^ used the IPIP^22^, which measures a combination of high conscientiousness and low agreeableness. They used poker card hands and participants should indicate their chances of winning after being presented anchors. For anchoring effects, they calculated rank order correlation across 140 trials of poker cards and found estiamtes to be correlate with their personality trait using Pearson correlations. Since this was across the sequence of 140 trials, this relationship was reasoned to show the ability of participants to learn during the experiment to be less influenced by anchor information.

Due to the different theoretical approaches, methods, sampling strategies, and analysis approaches of previous studies^14–19^, the question remains if, and how, personality might predict susceptibility to this cognitive bias. Personality traits describe individuals' characteristics, varying between participants. In its most elegant way, this could be done by measuring both biased and non-biased estimations. To circumvent this problem, some studies have used factorial designs to examine between-group differences^16^. To increase statistical power, ideally, the degree of the anchoring effect should be measured on the individual, within-subject level. Such an individual degree of the anchoring effect to personality measures is typically achieved by using several anchor questions, having counterbalanced high or low anchors, and comparing standardizing responses towards low and high anchored questions within-subject^10–12,23^. While this increases statistical power, this approach suffers from a different problem. It assumes that participants exhibit a similar knowledge for the different questions since studies have shown that differences in knowledge moderate anchor effects to a large extent^24^. While such knowledge effects might cancel out on the level of the whole sample, this approach is reasoned to be rather insensitive to detect relationships with personality traits on the individual level.

Taken together, while several personality traits have been related to the susceptibility to the anchoring effect, yet no clear relationship was observed in the different studies. Further, no study tested yet the evidence for the presence or *absence* of such an effect applying Bayesian statistics. In this study, we aimed at providing clarity by examining possible relationships between personality and the anchoring effect in a large sample consisting of more than 1000 participants. Participants were examined in the laboratory, examining their personality with a short version of the NEO-FFI^25^, and were asked four typical anchor questions with counterbalanced high or low anchors. To examine possible relationships, we made use of different analytic approaches, which incorporated both between- and—more importantly—within-subjects-analyses, which aimed to address the different described methodological problems. Importantly, by using Bayes Factors for all analyses, we are also able to quantify the evidence for the nonexistence of a relationship.

## Results

We used four counterbalanced (version A vs. B) anchor questions from different fields of knowledge. Hence, every participant was exposed to two questions with high and two with low anchors. The first question asked when Albert Einstein emigrated to the United States of America (anchor in version A: 1939, B: 1905). The second question asked when Leonardo da Vinci was born (A: 1698, B: 1391). The third question asked how old Mahatma Gandhi was when he died (A: 64 years, B: 79 years). The fourth question asked about the annual cubic milliliter of rain in the Sahara (A: 45 mm^3^, B: 90 mm^3^).

We used Bayes Factors for all analyses. A manipulation check of the given estimates was performed to test if the desired anchoring effects could be provoked. To replicate methodologies used in past studies^14–19^, we performed two different statistical tests to estimate relationships on a within-subject level. First, we aimed at examining individual anchor susceptibility by generating differences of z-transformed values for the respective low and high anchors, which then were correlated with Big Five scores by using Bayesian Pearson correlations. Secondly, to allow a more fine-grained test for a possible relationship, we tried to estimate the size of the anchoring effect per participant for each of the four questions. To this end, we ranked participants according to the absolute difference between the anchor values and participants' estimations and correlated these question-specific rank values and an aggregated rank score with personality traits using Bayesian Kendall’s Tau. For completeness, we also used statistical approaches of other previous studies which observed different relationships between personality and the susceptibility to the anchoring effect on the between-group level^16,18^, for which the detailed analyses are reported in the Supplementary Material.

### Manipulation check anchoring effect

Between-group analyses using Bayesian independent *t*-tests showed very strongevidence in favour of an anchoring effect for each question. Here, differences between anchors regarding Einsteins emigration (low anchor *M* = 1913, *SD* = 30, high anchor *M* = 1927, *SD* = 19,BF_10_ = 2.39^e+14^), da Vinci’s birth year (low anchor *M* = 1487, *SD* = 139, high anchor *M* = 1616, *SD* = 116, BF_10_ = 6.52^e+46^), Ghandi’sage at death (low anchor *M* = 71, *SD* = 10, high anchor *M* = 79, *SD* = 9,BF_10_ = 2.00^e+36^), and the average rain fall in the Sahara were found (low anchor *M* = 82, *SD* = 138, high anchor *M* = 129, *SD* = 173, BF_10_ = 5914). The balanced anchor versions enabled the comparison between high and low anchor estimates for each participant as a within-subject analysis. A Bayesian dependent paired t-test showed that evidence for an existing difference was 9.82^e+73^ times more likely than no differences. Thus, we could validate that high anchors elicit higher estimates than low anchors.

### Relationship between personality and anchor estimates

From the perspective that the relationship should betested on the within-subject level, we used two approaches to quantify within-subject anchor effects. Firstly, we correlated within-subject differences between z-transformed high and low anchors with Big Five personality traits. Bayesian Pearson correlations showed evidence for the absence of a relationship between anchor effects for all five personality traits. This evidence was, however, only anectodal for openness to experience (neuroticism: *r* = 0.036, BF_01_ = 13.35; extraversion: *r* = 0.007, BF_01_ = 24.51; openness to experience: *r* = − 0.067, BF_01_ = 2.69; agreeableness: *r* = 0.039, BF_01_ = 11.97; conscientiousness: *r* = − 0.015, BF_01_ = 22.35).

Secondly, we tested individual differences in participants' estimates biased towards the anchors. Therefore we first calculated the absolute differences between the given anchor values and participants' estimations (see Table 1, upper section). Then for each question, the rank information of anchor-estimate differences was related Big Five personality scores using Bayesian Kendall’s tau. In two cases (both for conscientiousness), rank differences for anchor estimates showed inconclusive evidence, however, differing in direction. Finally, we aggregated rank information of the four questions to generate a composite score of the anchor-bias. For this score, Bayesian Kendall’s tau showed evidence for the absence of a relationship with all personality traits (see Table 1, lower section).

**Table 1:** Descriptive differences between estimates and anchors and Bayesian Kendall’s tau correlations of rank differences and personality traits.

Question	Descriptives	Mean difference	Minimal/maximal difference	High/low anchors	Median high/low anchors	Anchoring index
Einstein migration	Years	17.13 (20.85)	0/355	1939/1905	1930/1920	0.29
DaVinci birth	Years	118.33 (101.87)	0/598	1698/1391	1620/1468	0.50
Gandhi age	Years	8.74 (6.16)	0/34	79/64	81/70	0.73
Sahara rain	mm^3^	75.36 (141.97)	1/1155	90/45	75/50	0.56

Question	Correlations	Neuro-ticism	Extra-version	Openness	Agreeable-ness	Conscien-tiousness
**Einstein migration**	Kendall’s tau	0.02	− 0.04	< .01	− 0.01	< − 0.01
	**BF**_**01**_	**14.30**	**5.78**	**24.00**	**21.30**	**22.28**
**DaVinci birth**	Kendall’s tau	0.03	< − 0.01	− 0.01	0.04	< − 0.01
	**BF**_**01**_	**8.29**	**23.06**	**19.06**	**5.90**	**24.01**
**Gandhi age**	Kendall’s tau	− 0.01	− 0.03	0.02	− 0.03	− 0.06
	**BF**_**01**_	**19.64**	**11.47**	**18.68**	**11.65**	0.65
**Sahara rain**	Kendall’s tau	0.02	< 0.01	− 0.01	0.01	0.05
	**BF**_**01**_	**16.32**	**23.86**	**21.40**	**19.53**	1.08
**Mean rank**	Kendall’s tau	0.03	− 0.03	< 0.01	< 0.01	< − 0.01
	**BF**_**01**_	**9.71**	**10.18**	**23.76**	**22.88**	**23.91**

BF_01_ displays how many times more likely the nonexistence of a relationship for the depicted Kendall tau correlation is. Bold fonts highlight BFs exhibiting at least moderate evidence against a relationship. Anchoring index values range from 0 (no anchor effects) to 1 (median estimates coincide with anchors).

### Control analyses: rank deviations relationships to z-score differences

While the deviations between anchor values and the estimates allowed a more fine-grained test for a possible relationship, this measure is yet uncommon and it is unclear how it relates to more commonly used methods. To this end, we correlated the mean rank information with the z-score difference between high and low anchors, showing extreme evidence for a relationship (Kendall’s tau = 0.343, BF_10_ = 3.09^e+55^). We also see such extreme evidence of a relationship three of the questions (Kendall’s tau between 0.184 and 0.283, BFs_10_ between 7.95^e+14^ and 1858^e+37^), while there is only strong evidence for the Sahara question (Kendall’s tau = 0.072, BF_10_ = 13.79). Further, to test if rank deviations reflect trait-like phenomena, we intercorrelated these rank information, showing for three out of six intercorrelations strong or extreme evidence for a relationship (see Table 2). For a relationship between the Davinci and Sahara question, an inconclusive relationship was found, while for the remaining two correlations, moderate to strong evidence against a relationship was seen (BF_01_ = 9.25 and 11.24).

**Table 2:** Rank intercorrelations using Bayesian Kendall's Tau.

	Sahara	Davinci	Einstein	Gandhi
**Sahara**				
Kendall's tau	–			
BF_10_	–			
**Davinci**				
Kendall's tau	0.058	–		
BF_10_	1.642	–		
**Einstein**				
Kendall's tau	0.104*	0.087*	–	
**BF**_**10**_	**6972**	**182**	–	
**Gandhi**				
Kendall's tau	− 0.026	0.075*	0.029	–
**BF**_**10**_	0.089^┼^	**23**	0.108	–

Bold fonts highlight BFs exhibiting at least strong evidence for a relationship. BF_01_ indicates evidence in favor of the null hypothesis (H0) and conversely BF_10_ evidence in favor of the alternative hypothesis (where BF_10_ = 1/BF_01_)

*BF_10_ > 10; ^┼^BF_01_ > 10.

## Discussion

In this study, we tested if, and which, personality traits are related to a higher susceptibility to the anchoring effect. To this end, we used common methods of quantifying the anchoring effect and linking anchoring susceptibility to personality traits, as well as developed new measures to find a link between the two constructs. However, while previous studies reported multiple but conflicting relationships, we show evidence against a systematic influence of personality traits on the susceptibility towards anchoring effects in common anchoring designs. It remains unclear if the commonly measured anchoring effect is simply not reflecting a trait-like construct, or if it is linked to individual trait measures other than the established Big Five personality traits. Nevertheless, if a link to personality existed, this would most likely have become apparent as we examined a rather large sample of approximately 1000 participants for which our manipulation resulted in a typical anchoring effect both when applying a between- and a within-subject approach^8,12^ and all questions effectively shifted the median response towards the anchor^26^. Further, the collected NEO-personality scores closely resembled those described in normative samples^27–29^.

Importantly, we reasoned that the anchoring effect should be measured on an individual level to detect potential personality effects. Following this idea, we utilized z-transformations^10–12^, comparing the differences between high and low anchors. Here, for each trait, evidence for the absence of a relationship between Big Five traits and the anchoring effect was found. Further, we reasoned that participants who are more susceptible to the anchoring effect might orient their responses more strongly towards the given anchor. Thus, we calculated the absolute differences between the given anchor values and participants' estimations, ranked each participant, and finally aggregated the rank information across the four questions, showing evidence against a relationship with all Big Five traits. Our findings are in line with a highly interesting and the most recent contribution to the ongoing debate on relationships between the anchoring effect and the Big Five personality traits by Cheek and Norem^19^. This study coincidentally used a similar approach available to us during the resubmission of this report. In this study, a large sample of 1000 participants was examined online with six different anchor questions and the authors examined the full NEO-PI-R questionnaire. Personality scores and anchoring values were correlated using inferential statistics. Interestingly, this study likewise did not reveal significant relationships when correcting for multiple comparisons. Given the discussed heterogeneity in the approaches used in prior studies, and thus the multiple analytic strategies we incorporated in our study, it seems reasonable to limit comparison to the most recent study^19^. When focusing on this similar composite score, we see highly similar effect sizes (*rs* between 0.01 and 0.04). The only marginal difference is related to conscientiousness, where we found no relationship (*r* < 0.01) but Cheek and Norem^19^ report a correlation of *r* = 0.09. This might be either due to the better capture of conscientiousness by Cheek and Norem who used the NEO-PI-R inventory or due to a limited range of conscientiousness scores in our sample, as this personality trait can be assumed to have a more similar distribution within medical students. However, we see at least no strong discrepancy regarding conscientiousness between our sample and the normative sample of the used short version of the NEO-FFI^25^.

For completeness, we also report between-subject analysis results to provide comparable results as in other studies^16,18^ (see Supplementary Materials). With the caveat that such approaches might result in spurious findings^30^, these Bayesian ANOVAs were at best inconclusive in three cases (for neuroticism, for extraversion, and openness, but in each case in one out of for questions). This is similar to the observation by Furnham and colleagues^18^, who emphasized that a significant inferential relationship was observed only for one out of four questions. Likewise, if performing frequentist analyses, for single questions and traits, we could find in our data support for different reported relationships. For example, we could show that anchor effects increase with higher levels of openness^14,16^. We could also show higher levels of conscientiousness related to anchor effects as observed for the Sahara question^14,15^, or, just the opposite, with lower levels of conscientiousness (Gandhi question). Since Bayesian statistics enable us to provide also evidence against the null hypothesis^31,32^, we thus find evidence against a systematic relationship. A methodological problem here pertains to the fact that each of the multiple reported relationships exhibits high face-validity^14–18^ since both concepts have broad applicability. However, importantly, we can show that there is no systematic pattern of relationships across questions or traits, as the experimental design allowed different analysis strategies.

### Constraints on generality

We can provide an answer to the question if there is a link between personality and susceptibility to the anchoring effect using a common methodology. It has been discussed that self-reported personality is related to the cultural background, and for example, cultural differences, rather than self-reported conscientiousness, predicted behavior^33^. Besides inconclusive evidence on that point^34^, our homogenous sample of predominantly young German medical students can exclude that cross-cultural differences account for the absence of a relationship. But this homogenous sample does not rule out the option that personality traits modulate the anchoring effect in other populations. Further, there might be relationships when using different anchoring designs, including other anchoring variants or questions, self-generated anchors^35^, numerical anchoring^36^, or sequential anchoring^37^. Besides, while anchoring effects are typically examined on knowledge questions, we might find rather a relationship to personality scores in other domains (e.g. when examining topics with higher social desirability), or when put into a different context (e.g. high anchors in negotiations might work better on more agreeable people). It should be noted, however, that reported relationships of personality and the anchoring effects used highly similar knowledge questions^14,16,18^. Related to this, despite the number of studies reporting relationships, due to the disagreement of a specific relationship, we could not specify prior assumptions to our Bayesian analyses. Finally, it cannot be completely ruled out that anchoring effects, on the between- or within-subject level might simply not reflect a reliable trait-like construct, which would prevent linking it to other individual traits due to a mere lack of construct-validity.

### Outlook

**Figure 1:**
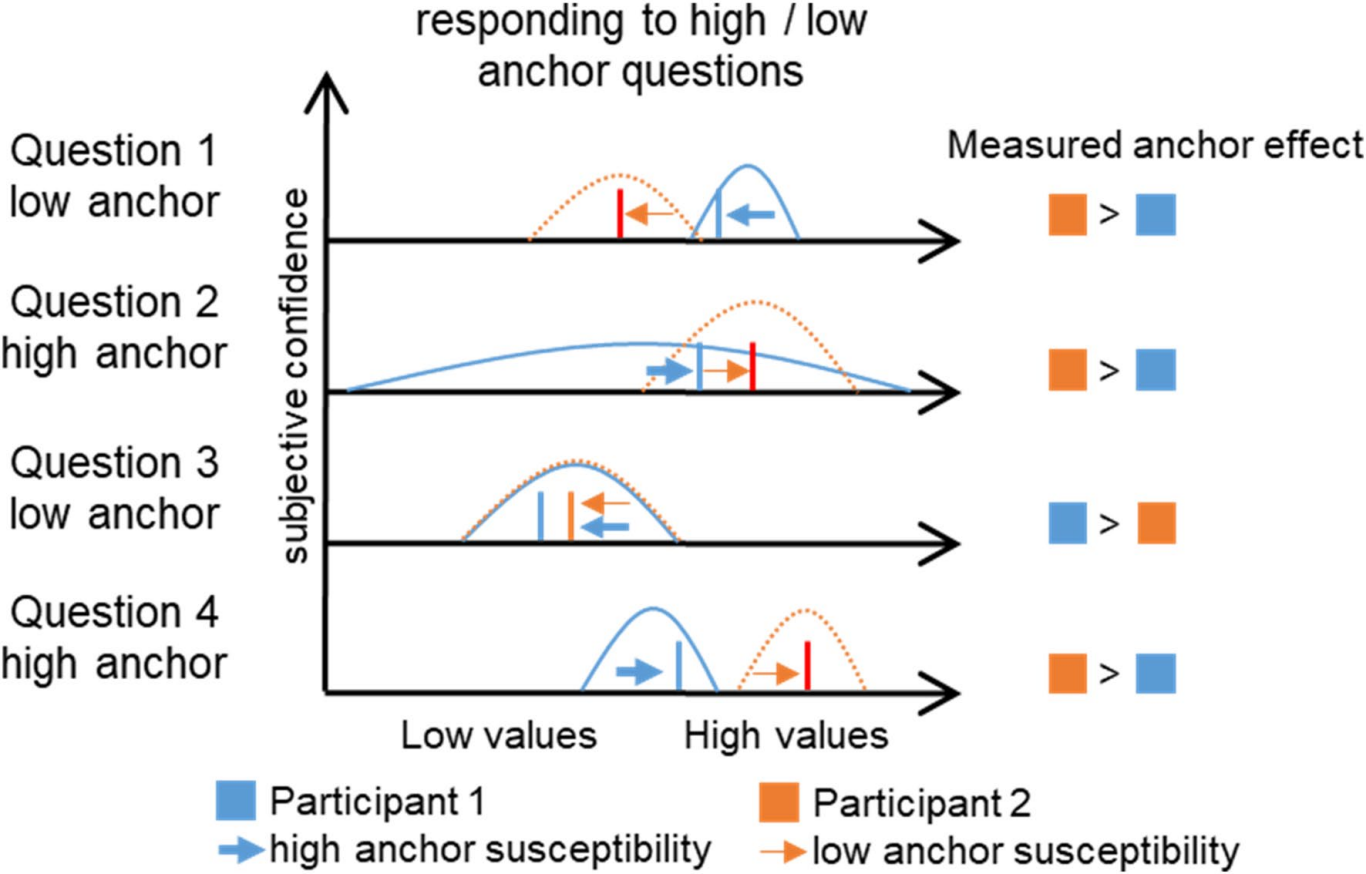
Schematic illustration of problems to quantify anchor effects on the individual level. Hypothetical underlying uncertainties for two persons (blue and orange) show different possible ranges of estimates for a given question. Bars illustrate their given estimates, which are shifted by anchors within their range of possible answers due to uncertainty. The blue participant is actually much more susceptible to anchors than the orange participants, but a stronger anchor effect (lower estimate for low anchor) is only observed for question 3, when they hypothetically have identical knowledge and thus exhibit the same range of plausible values (Question 3).

To address these limiting aspects, the crucial next step to test anchor-susceptibility should focus on the reliability of individual anchoring susceptibility, i.e. is to gather evidence that this is indeed a trait-like cognitive style. Further, studies have shown that differences in knowledge moderate anchor effects to a large extent^24^. It is furthermost likely that not only uncertainty but other influences on individual decision heuristics exist, and thus the anchoring effect should be ideally quantified as a deviation from the unbiased rating (see Fig. 1). While the anchoring effect depends on (at least some) uncertainty about the true answer to a given question^8^, a similar uncertainty might still lead to many different idiosyncratic reference points. For example, for guessing the average mm^3^ rain in the Sahara per year, some participants might use the average mm^3^ rain in their country as a reference, others might use zero as a reference, and some might use the anchor itself (see Fig. 1). Importantly, a low estimate of the length of the Mississippi and a high estimate of the population size of Belgium might reflect a different knowledge, different reference points, or simply depict erroneous certainty about the true answer. While heuristics might be diverse, at least reference points might be shared in some cases (e.g. for guessing historic events, some significant and common-known dates could pose reference points for most participants). Interestingly, it has been reported that participants have an implicit range of plausible results^10^. Here, the anchor shifts where participants’ responses towards the anchor within that range of possible answers^10^. Assessing these ranges might help to understand relationships between personality and the anchoring effect. Other promising approaches would be to capture if certain facets of the NEO-PI-R^3^ are linked rather to normative components of personality judgments since these are more prone to social desirability.

## Conclusion

To conclude, personality is a broad predictor of human response behavior^1,2^, and individual characteristics are likely important for the susceptibility to the anchoring effect^13^. However, for a common anchor design on knowledge questions, we provide evidence against a systematic relationship between personality traits and the anchoring effect.

## Methods

### Participants

In total, 1044 participants were recruited at the University of Muenster. After removal of outliers (estimates above or below 2.5 standard deviations of the mean score, incomplete/missing estimates, and negative estimates due to logical reasons), the final sample eventually consisted of 992 participants. Participants gave informed consent and completed the experiment as part of their teaching requirements in the second semester at the University of Muenster. We did not collect nor use any personal data for further analyses. However, the respective distribution of the sample matched the distribution of medical students in the second semester at the University of Muenster (~ 60% female and ~ 22 years of age on average). The study was granted ethics approval by the ethics committee of the German Society for Psychology (Deutsche Gesellschaft für Psychologie, DGPS e.v., https://zwpd.transmit.de/zwpd-dienstleistungen/zwpd-ethikkommission/). All methods were performed in accordance with the guidelines and regulations at the University of Muenster.

### Measures

We used the short version of the NEO-FFI^25^, which is a personality questionnaire consisting of 30 items summed up to the Big Five personality factors neuroticism, extraversion, openness for experience, agreeableness, and conscientiousness. The items of the NEO-FFI are scaled as a Likert-type scale, with five possible answers (*strongly disagree*–*strongly agree*). While we used the short version, we observe the same intercorrelations between personality trait of the NEO-FFI as reported in the German manual of the NEO-FFI^27^: Correlation between neuroticism and extraversion − 0.31 in our sample vs. − 0.33 in the manual; correlation between agreeableness and extraversion 0.17 vs. 0.16; correlation between conscientiousness and neuroticism − 0.30 vs. − 0.31. Further, for this 30-item short version, a normative study show highly similar mean scores and Cronbach’s alpha as measure of internal consistency^25^: Neuroticism (*M*_*our sample*_ = 1.39, *SD* = 0.78; Cronbach’s α = 0.83 vs. *M*_*normative study*_ = 1.52, *SD* = 0.77; Cronbach’s α = 0.81), extraversion (*M*_*our sample*_ = 2.54, SD = 0.57; Cronbach’s α = 0.70 vs. *M* = 2.28, *SD* = 0.62; Cronbach’s α = 0.72), openness to experience (*M*_*our sample*_ = 2.60, *SD* = 0.77; Cronbach’s α = 0.79 vs. *M* = 2.04, *SD* = 0.64; Cronbach’s α = 0.67), agreeableness (*M*_*our sample*_ = 2.89, *SD* = 0.64; Cronbach’s α = 0.72 vs. *M* = 2.79, *SD* = 0.65; Cronbach’s α = 0.75), and conscientiousness (*M*_*our sample*_ = 3.08, *SD* = 0.58; Cronbach’s α = 0.76 vs. *M* = 2.96, *SD* = 0.62; Cronbach’s α = 0.78).

We used four anchor questions, counterbalanced two with high and two with low anchors. The first question asked when Albert Einstein emigrated to the United States of America (high anchor 1939, low anchor 1905). The second question asked when Leonardo da Vinci was born (high anchor 1698, low anchor 1391). The third question asked how old Mahatma Gandhi was when he died (high anchor 79 years, low anchor 64 years). Finally, the fourth question asked about the annual cubic milliliter of rain in the Sahara (high anchor 90 mm^3^, low anchor 45 mm^3^). Each anchor was presented as a question: e.g. Sahara question, high anchor "Do you think the annual cubic milliliter of rain in the Sahara is higher or lower than 90mm^3^?". Then, the participant had to give his/her estimate. We calculated the anchoring index following the proposed method by Jancowitz and Kahneman^26^, showing across the median subject moved halfway toward the anchor across the four questions, showing the least effects for the question on Einstein's emigration.

### Procedure

All participants attended the experiment as part of empirical research training. During the training at the research institute, they were verbally instructed to fill in two questionnaires. Subsequently, instructions were given on a computer to avoid instructor effects. Participants first completed the 30-item short German version of the NEO-FFI^3,27^, and then answered four estimation questions for each of which an anchor was set (regarding the anchoring effect). The question set existed in two versions (A and B), each with two high and with two low anchored questions (in reversed order). Participants were randomly assigned to the anchor version A (resulting in *N* = 500) or B (*N* = 492).

### Data analyses

We used Bayes Factors for all analyses. We specified the null hypothesis as a point-null prior (i.e., standardized effect size *δ* = 0), whereas the alternative hypothesis was defined as a Jeffreys-Zellner-Siow (*JZS*) prior, i.e., a folded Cauchy distribution centered around *δ* = 0 with scaling factors of *r* = 0.707. This scaling factor assumes a roughly normal distribution. To assign verbal labels to the strength of evidence, we followed the taxonomy suggested by Jeffreys^38^, labeling Bayes Factors with a BF_10_ of 1 as no evidence, BF_10_ between 1 and 3 as anecdotal evidence, 3–10 as moderate evidence, 10–30 as strong evidence, 30–100 as very strong evidence, and larger BFs as extreme evidence in favor of the alternative hypothesis. BF_10_ indicates evidence in favor of the alternative hypotheses, while the reverse BF_01_ indicates evidence in favour of the null hypotheses (i.e. no differences).

For the manipulation check, Bayesian independent *t*-tests were calculated for between-group and Bayesian dependent *t*-tests for within-subject analyses. For testing the relationship between personality and the susceptibility to the anchoring effect, we aimed at examining anchor susceptibility on the individual level using within-subject analyses. In a first step, we generated z-transformed values for the respective low and high anchors, averaged for the two low and high anchor questions. These resulting within-subject differences were correlated with Big Five scores by using Bayesian Pearson correlations. For the second analysis, we ranked participants according to the absolute difference between the anchor values and participants' estimations and correlated this rank value with personality traits using Bayesian Kendall’s Tau. We further correlated an aggregated rank score (S) across the four questions with personality traits:

$$ {\text{S}}_{{{\text{anchor}}}} = {\text{ Mean }}\left( {{\text{rank }}\left( {{\text{d}}_{{\text{question i}}} } \right) \, + \, \cdots \, + {\text{ rank }}\left( {{\text{d}}_{{\text{question I}}} } \right)} \right) $$

with, d_question i_ = │x _question i_ − a _question i_│, x_question i_ = estimation, a_question i_ = given anchor, i … I = running number of questions.

## Supplementary Information

Supplementary Information.

## Data Availability

All data is available on the Open Science Framework (https://osf.io/r48wq/).
