# Supplementary material for: Bayes Factors show evidence against systematic relationships between the anchoring effect and the Big Five personality traits
Source: Sci Rep. 2021 Mar 29;11:7021. doi: 10.1038/s41598-021-86429-2 (PMC8007589; doi:10.1038/s41598-021-86429-2)
Supplement: Supplementary file 1 — Supplementary Information. [file 41598_2021_86429_MOESM1_ESM.docx]

**Bayes Factors show evidence against systematic relationships between the anchoring effect and the Big Five personality traits**

- **Supplementary Materials -**

Sebastian Schindler^1,2┼^, Jan Querengässer^3*┼^, Maximilian Bruchmann^1,2^, Nele Johanna Bögemann^1^, Robert Moeck^1^, and Thomas Straube^1,2^

^1^ Institute of Medical Psychology and Systems Neuroscience, University of Muenster

^2^ Otto Creutzfeldt Center for Cognitive and Behavioral Neuroscience, University of Muenster

^3^University of Konstanz, Department of Psychology

^┼^ Equal contributing authors

* Corresponding author

**Correspondence address**

Institute of Medical Psychology and Systems Neuroscience

University of Muenster

Von-Esmarch-Str. 52, D-48149 Muenster, Germany

e-mail: [sebastian.schindler@ukmuenster.de](mailto:sebastian.schindler@ukmuenster.de)

**Between-group analyses on personality-anchor susceptibility relationships**

To use similar approaches reported in previous studies ^16,18^, we also used a between-group analysis. We divided the sample into quartiles for each personality trait and tested interaction effects using Bayesian ANOVA models. For assessing the relationship between personality and anchor estimates, we firstly chose a between-factorial design by dividing the sample into quartiles for each Big Five trait (see Supplementary Table S1). We then tested for interaction effects by using Bayesian ANOVA models (see Supplementary Table S2). Results showed evidence for the absence of interactions, while in three cases inconclusive results were found. This lack of clarifying pertains to the question about Einstein’s emigration for neuroticism, the question about Gandhi's age for extraversion, and the question about the average rain in the Sahara for openness.

| **Supplementary Table S1. Descriptive anchor estimates for each personality factor across quartiles** |
| --- |

| Neuroticism | | Q1 (0-25%) | Q2 (25-50%) | Q3 (50-75%) | Q 4 (75-100%) |
| --- | --- | --- | --- | --- | --- |
| Question Einstein | Low (B) | 1915  (*SD*=21;*N*=112) | 1917  (*SD*=19;*N*=132) | 1907  (*SD*=44;*N*=124) | 1913  (*SD*=28; *N* =124) |
|  | High(A) | 1925  (*SD*=18; *N*=136) | 1927  (*SD*=17; *N*=116) | 1928  (*SD*=21; *N*=124) | 1927  (*SD*=20; *N*=124) |
| Question da Vinci | Low (B) | 1485  (*SD*=143; *N*=112) | 1506  (*SD*=138; *N*=132) | 1479  (*SD*=149; *N*=124) | 1477  (*SD*=127; *N*=124) |
|  | High (A) | 1595  (*SD*=121; *N*=136) | 1624  (*SD*=94; *N*=116) | 1621  (*SD*=124; *N*=124) | 1624  (*SD*=119; *N*=124) |
| Question Gandhi | Low (A) | 70  (*SD*=9; *N*=136) | 70  (*SD*=10; *N*=116) | 72  (*SD*=11; *N*=124) | 71  (*SD*=12; *N*=124) |
|  | High (B) | 78  (*SD*=9; *N*=112) | 79  (*SD*=9; *N*=132) | 79  (*SD*=8; *N*=124) | 79  (*SD*=9; *N*=124) |
| Question Sahara | Low (A) | 82  (*SD*=135; *N*=136) | 58  (*SD*=64; *N*=116) | 103  (*SD*=182; *N*=124) | 82  (*SD*=138; *N*=124) |
|  | High (B) | 131  (*SD*=182; *N*=112) | 122  (*SD*=150; *N*=132) | 135  (*SD*=179;*N*=124) | 131  (*SD*=183; *N*=124) |
| **Extraversion** | | **Q1 (0-25%)** | **Q2 (25-50%)** | **Q3 (50-75%)** | **Q 4 (75-100%)** |
| Question Einstein | Low (B) | 1912  (*SD*=28; *N*=127)    Abhängige Variable:anker_einstein_estimate  anker_version E_Quartiles Mittelwert Std.-Abweichung N  A 1,00 1926,83 19,172 121  2,00 1927,59 16,878 128  3,00 1926,44 19,463 128  4,00 1925,54 19,492 123  Gesamt 1926,60 18,728 500  B 1,00 1912,06 28,386 127  2,00 1911,31 30,072 120  3,00 1913,48 38,219 120  4,00 1914,73 21,120 125  Gesamt 1912,90 29,906 492  Gesamt 1,00 1919,26 25,384 248  2,00 1919,71 25,468 248  3,00 1920,17 30,669 248  4,00 1920,09 20,998 248  Gesamt 1919,81 25,821 992  Deskriptive Statistiken  Abhängige Variable:anker_einstein_estimate  anker_version E_Quartiles Mittelwert Std.-Abweichung N  A 1,00 1926,83 19,172 121  2,00 1927,59 16,878 128  3,00 1926,44 19,463 128  4,00 1925,54 19,492 123  Gesamt 1926,60 18,728 500  B 1,00 1912,06 28,386 127  2,00 1911,31 30,072 120  3,00 1913,48 38,219 120  4,00 1914,73 21,120 125  Gesamt 1912,90 29,906 492  Gesamt 1,00 1919,26 25,384 248  2,00 1919,71 25,468 248  3,00 1920,17 30,669 248  4,00 1920,09 20,998 248  Gesamt 1919,81 25,821 992 | 1911  (*SD*=30; *N*=120) | 1913  (*SD*=38; *N*=120) | 1915  (*SD*=21; *N*=125) |
|  | High(A) | 1927  (*SD*=19; *N*=121) | 1928  (*SD*=17; *N*=128) | 1926  (*SD*=19; *N*=128) | 1926  (*SD*=19; *N*=123) |
| Question da Vinci | Low (B) | 1496  (*SD*=123; *N*=127)    Abhängige Variable:anker_einstein_estimate  anker_version E_Quartiles Mittelwert Std.-Abweichung N  A 1,00 1926,83 19,172 121  2,00 1927,59 16,878 128  3,00 1926,44 19,463 128  4,00 1925,54 19,492 123  Gesamt 1926,60 18,728 500  B 1,00 1912,06 28,386 127  2,00 1911,31 30,072 120  3,00 1913,48 38,219 120  4,00 1914,73 21,120 125  Gesamt 1912,90 29,906 492  Gesamt 1,00 1919,26 25,384 248  2,00 1919,71 25,468 248  3,00 1920,17 30,669 248  4,00 1920,09 20,998 248  Gesamt 1919,81 25,821 992  Deskriptive Statistiken  Abhängige Variable:anker_einstein_estimate  anker_version E_Quartiles Mittelwert Std.-Abweichung N  A 1,00 1926,83 19,172 121  2,00 1927,59 16,878 128  3,00 1926,44 19,463 128  4,00 1925,54 19,492 123  Gesamt 1926,60 18,728 500  B 1,00 1912,06 28,386 127  2,00 1911,31 30,072 120  3,00 1913,48 38,219 120  4,00 1914,73 21,120 125  Gesamt 1912,90 29,906 492  Gesamt 1,00 1919,26 25,384 248  2,00 1919,71 25,468 248  3,00 1920,17 30,669 248  4,00 1920,09 20,998 248  Gesamt 1919,81 25,821 992 | 1491  (*SD*=143; *N*=120) | 1482  (*SD*=159; *N*=120) | 1479  (*SD*=134; *N*=125) |
|  | High (A) | 1609  (*SD*=106; *N*=121) | 1617  (*SD*=125; *N*=128) | 1613  (*SD*=117; *N*=128) | 1625  (*SD*=115; *N*=123) |
| Question Gandhi | Low (A) | 70  (*SD*=11; *N*=121) | 70  (*SD*=10; *N*=128) | 69  (*SD*=10; *N*=128) | 73  (*SD*=10; *N*=123) |
|  | High (B) | 79  (*SD*=9; *N*=127) | 79  (*SD*=8; *N*=120) | 79  (*SD*=8; *N*=120) | 78  (*SD*=10; *N*=125) |
| Question Sahara | Low (A) | 84  (*SD*=149; *N*=121) | 94  (*SD*=190; *N*=128) | 61  (*SD*=59; *N*=128) | 88  (*SD*=118; *N*=123) |
|  | High (B) | 124  (*SD*=184; *N*=127) | 121  (*SD*=156; *N*=120) | 149  (*SD*=195; *N*=120) | 124  (*SD*=155; *N*=125) |
| **Openness** | | **Q1 (0-25%)** | **Q2 (25-50%)** | **Q3 (50-75%)** | **Q 4 (75-100%)** |
| Question Einstein | Low (B) | 1908  (*SD*=44; *N*=125)    Abhängige Variable:anker_einstein_estimate  anker_version E_Quartiles Mittelwert Std.-Abweichung N  A 1,00 1926,83 19,172 121  2,00 1927,59 16,878 128  3,00 1926,44 19,463 128  4,00 1925,54 19,492 123  Gesamt 1926,60 18,728 500  B 1,00 1912,06 28,386 127  2,00 1911,31 30,072 120  3,00 1913,48 38,219 120  4,00 1914,73 21,120 125  Gesamt 1912,90 29,906 492  Gesamt 1,00 1919,26 25,384 248  2,00 1919,71 25,468 248  3,00 1920,17 30,669 248  4,00 1920,09 20,998 248  Gesamt 1919,81 25,821 992  Deskriptive Statistiken  Abhängige Variable:anker_einstein_estimate  anker_version E_Quartiles Mittelwert Std.-Abweichung N  A 1,00 1926,83 19,172 121  2,00 1927,59 16,878 128  3,00 1926,44 19,463 128  4,00 1925,54 19,492 123  Gesamt 1926,60 18,728 500  B 1,00 1912,06 28,386 127  2,00 1911,31 30,072 120  3,00 1913,48 38,219 120  4,00 1914,73 21,120 125  Gesamt 1912,90 29,906 492  Gesamt 1,00 1919,26 25,384 248  2,00 1919,71 25,468 248  3,00 1920,17 30,669 248  4,00 1920,09 20,998 248  Gesamt 1919,81 25,821 992 | 1912  (*SD*=27; *N*=127)    Abhängige Variable:anker_einstein_estimate  anker_version E_Quartiles Mittelwert Std.-Abweichung N  A 1,00 1926,83 19,172 121  2,00 1927,59 16,878 128  3,00 1926,44 19,463 128  4,00 1925,54 19,492 123  Gesamt 1926,60 18,728 500  B 1,00 1912,06 28,386 127  2,00 1911,31 30,072 120  3,00 1913,48 38,219 120  4,00 1914,73 21,120 125  Gesamt 1912,90 29,906 492  Gesamt 1,00 1919,26 25,384 248  2,00 1919,71 25,468 248  3,00 1920,17 30,669 248  4,00 1920,09 20,998 248  Gesamt 1919,81 25,821 992  Deskriptive Statistiken  Abhängige Variable:anker_einstein_estimate  anker_version E_Quartiles Mittelwert Std.-Abweichung N  A 1,00 1926,83 19,172 121  2,00 1927,59 16,878 128  3,00 1926,44 19,463 128  4,00 1925,54 19,492 123  Gesamt 1926,60 18,728 500  B 1,00 1912,06 28,386 127  2,00 1911,31 30,072 120  3,00 1913,48 38,219 120  4,00 1914,73 21,120 125  Gesamt 1912,90 29,906 492  Gesamt 1,00 1919,26 25,384 248  2,00 1919,71 25,468 248  3,00 1920,17 30,669 248  4,00 1920,09 20,998 248  Gesamt 1919,81 25,821 992 | 1915  (*SD*=21; *N*=127)    Abhängige Variable:anker_einstein_estimate  anker_version E_Quartiles Mittelwert Std.-Abweichung N  A 1,00 1926,83 19,172 121  2,00 1927,59 16,878 128  3,00 1926,44 19,463 128  4,00 1925,54 19,492 123  Gesamt 1926,60 18,728 500  B 1,00 1912,06 28,386 127  2,00 1911,31 30,072 120  3,00 1913,48 38,219 120  4,00 1914,73 21,120 125  Gesamt 1912,90 29,906 492  Gesamt 1,00 1919,26 25,384 248  2,00 1919,71 25,468 248  3,00 1920,17 30,669 248  4,00 1920,09 20,998 248  Gesamt 1919,81 25,821 992  Deskriptive Statistiken  Abhängige Variable:anker_einstein_estimate  anker_version E_Quartiles Mittelwert Std.-Abweichung N  A 1,00 1926,83 19,172 121  2,00 1927,59 16,878 128  3,00 1926,44 19,463 128  4,00 1925,54 19,492 123  Gesamt 1926,60 18,728 500  B 1,00 1912,06 28,386 127  2,00 1911,31 30,072 120  3,00 1913,48 38,219 120  4,00 1914,73 21,120 125  Gesamt 1912,90 29,906 492  Gesamt 1,00 1919,26 25,384 248  2,00 1919,71 25,468 248  3,00 1920,17 30,669 248  4,00 1920,09 20,998 248  Gesamt 1919,81 25,821 992 | 1917  (*SD*=19; *N*=113)    Abhängige Variable:anker_einstein_estimate  anker_version E_Quartiles Mittelwert Std.-Abweichung N  A 1,00 1926,83 19,172 121  2,00 1927,59 16,878 128  3,00 1926,44 19,463 128  4,00 1925,54 19,492 123  Gesamt 1926,60 18,728 500  B 1,00 1912,06 28,386 127  2,00 1911,31 30,072 120  3,00 1913,48 38,219 120  4,00 1914,73 21,120 125  Gesamt 1912,90 29,906 492  Gesamt 1,00 1919,26 25,384 248  2,00 1919,71 25,468 248  3,00 1920,17 30,669 248  4,00 1920,09 20,998 248  Gesamt 1919,81 25,821 992  Deskriptive Statistiken  Abhängige Variable:anker_einstein_estimate  anker_version E_Quartiles Mittelwert Std.-Abweichung N  A 1,00 1926,83 19,172 121  2,00 1927,59 16,878 128  3,00 1926,44 19,463 128  4,00 1925,54 19,492 123  Gesamt 1926,60 18,728 500  B 1,00 1912,06 28,386 127  2,00 1911,31 30,072 120  3,00 1913,48 38,219 120  4,00 1914,73 21,120 125  Gesamt 1912,90 29,906 492  Gesamt 1,00 1919,26 25,384 248  2,00 1919,71 25,468 248  3,00 1920,17 30,669 248  4,00 1920,09 20,998 248  Gesamt 1919,81 25,821 992 |
|  | High(A) | 1924  (*SD*=21; *N*=123)    Abhängige Variable:anker_einstein_estimate  anker_version E_Quartiles Mittelwert Std.-Abweichung N  A 1,00 1926,83 19,172 121  2,00 1927,59 16,878 128  3,00 1926,44 19,463 128  4,00 1925,54 19,492 123  Gesamt 1926,60 18,728 500  B 1,00 1912,06 28,386 127  2,00 1911,31 30,072 120  3,00 1913,48 38,219 120  4,00 1914,73 21,120 125  Gesamt 1912,90 29,906 492  Gesamt 1,00 1919,26 25,384 248  2,00 1919,71 25,468 248  3,00 1920,17 30,669 248  4,00 1920,09 20,998 248  Gesamt 1919,81 25,821 992  Deskriptive Statistiken  Abhängige Variable:anker_einstein_estimate  anker_version E_Quartiles Mittelwert Std.-Abweichung N  A 1,00 1926,83 19,172 121  2,00 1927,59 16,878 128  3,00 1926,44 19,463 128  4,00 1925,54 19,492 123  Gesamt 1926,60 18,728 500  B 1,00 1912,06 28,386 127  2,00 1911,31 30,072 120  3,00 1913,48 38,219 120  4,00 1914,73 21,120 125  Gesamt 1912,90 29,906 492  Gesamt 1,00 1919,26 25,384 248  2,00 1919,71 25,468 248  3,00 1920,17 30,669 248  4,00 1920,09 20,998 248  Gesamt 1919,81 25,821 992 | 1927  (*SD*=20; *N*=121)    Abhängige Variable:anker_einstein_estimate  anker_version E_Quartiles Mittelwert Std.-Abweichung N  A 1,00 1926,83 19,172 121  2,00 1927,59 16,878 128  3,00 1926,44 19,463 128  4,00 1925,54 19,492 123  Gesamt 1926,60 18,728 500  B 1,00 1912,06 28,386 127  2,00 1911,31 30,072 120  3,00 1913,48 38,219 120  4,00 1914,73 21,120 125  Gesamt 1912,90 29,906 492  Gesamt 1,00 1919,26 25,384 248  2,00 1919,71 25,468 248  3,00 1920,17 30,669 248  4,00 1920,09 20,998 248  Gesamt 1919,81 25,821 992  Deskriptive Statistiken  Abhängige Variable:anker_einstein_estimate  anker_version E_Quartiles Mittelwert Std.-Abweichung N  A 1,00 1926,83 19,172 121  2,00 1927,59 16,878 128  3,00 1926,44 19,463 128  4,00 1925,54 19,492 123  Gesamt 1926,60 18,728 500  B 1,00 1912,06 28,386 127  2,00 1911,31 30,072 120  3,00 1913,48 38,219 120  4,00 1914,73 21,120 125  Gesamt 1912,90 29,906 492  Gesamt 1,00 1919,26 25,384 248  2,00 1919,71 25,468 248  3,00 1920,17 30,669 248  4,00 1920,09 20,998 248  Gesamt 1919,81 25,821 992 | 1925  (*SD*=19; *N*=121)    Abhängige Variable:anker_einstein_estimate  anker_version E_Quartiles Mittelwert Std.-Abweichung N  A 1,00 1926,83 19,172 121  2,00 1927,59 16,878 128  3,00 1926,44 19,463 128  4,00 1925,54 19,492 123  Gesamt 1926,60 18,728 500  B 1,00 1912,06 28,386 127  2,00 1911,31 30,072 120  3,00 1913,48 38,219 120  4,00 1914,73 21,120 125  Gesamt 1912,90 29,906 492  Gesamt 1,00 1919,26 25,384 248  2,00 1919,71 25,468 248  3,00 1920,17 30,669 248  4,00 1920,09 20,998 248  Gesamt 1919,81 25,821 992  Deskriptive Statistiken  Abhängige Variable:anker_einstein_estimate  anker_version E_Quartiles Mittelwert Std.-Abweichung N  A 1,00 1926,83 19,172 121  2,00 1927,59 16,878 128  3,00 1926,44 19,463 128  4,00 1925,54 19,492 123  Gesamt 1926,60 18,728 500  B 1,00 1912,06 28,386 127  2,00 1911,31 30,072 120  3,00 1913,48 38,219 120  4,00 1914,73 21,120 125  Gesamt 1912,90 29,906 492  Gesamt 1,00 1919,26 25,384 248  2,00 1919,71 25,468 248  3,00 1920,17 30,669 248  4,00 1920,09 20,998 248  Gesamt 1919,81 25,821 992 | 1930  (*SD*=15; *N*=135)    Abhängige Variable:anker_einstein_estimate  anker_version E_Quartiles Mittelwert Std.-Abweichung N  A 1,00 1926,83 19,172 121  2,00 1927,59 16,878 128  3,00 1926,44 19,463 128  4,00 1925,54 19,492 123  Gesamt 1926,60 18,728 500  B 1,00 1912,06 28,386 127  2,00 1911,31 30,072 120  3,00 1913,48 38,219 120  4,00 1914,73 21,120 125  Gesamt 1912,90 29,906 492  Gesamt 1,00 1919,26 25,384 248  2,00 1919,71 25,468 248  3,00 1920,17 30,669 248  4,00 1920,09 20,998 248  Gesamt 1919,81 25,821 992  Deskriptive Statistiken  Abhängige Variable:anker_einstein_estimate  anker_version E_Quartiles Mittelwert Std.-Abweichung N  A 1,00 1926,83 19,172 121  2,00 1927,59 16,878 128  3,00 1926,44 19,463 128  4,00 1925,54 19,492 123  Gesamt 1926,60 18,728 500  B 1,00 1912,06 28,386 127  2,00 1911,31 30,072 120  3,00 1913,48 38,219 120  4,00 1914,73 21,120 125  Gesamt 1912,90 29,906 492  Gesamt 1,00 1919,26 25,384 248  2,00 1919,71 25,468 248  3,00 1920,17 30,669 248  4,00 1920,09 20,998 248  Gesamt 1919,81 25,821 992 |
| Question da Vinci | Low (B) | 1493  (*SD*=141; *N*=125)    Abhängige Variable:anker_einstein_estimate  anker_version E_Quartiles Mittelwert Std.-Abweichung N  A 1,00 1926,83 19,172 121  2,00 1927,59 16,878 128  3,00 1926,44 19,463 128  4,00 1925,54 19,492 123  Gesamt 1926,60 18,728 500  B 1,00 1912,06 28,386 127  2,00 1911,31 30,072 120  3,00 1913,48 38,219 120  4,00 1914,73 21,120 125  Gesamt 1912,90 29,906 492  Gesamt 1,00 1919,26 25,384 248  2,00 1919,71 25,468 248  3,00 1920,17 30,669 248  4,00 1920,09 20,998 248  Gesamt 1919,81 25,821 992  Deskriptive Statistiken  Abhängige Variable:anker_einstein_estimate  anker_version E_Quartiles Mittelwert Std.-Abweichung N  A 1,00 1926,83 19,172 121  2,00 1927,59 16,878 128  3,00 1926,44 19,463 128  4,00 1925,54 19,492 123  Gesamt 1926,60 18,728 500  B 1,00 1912,06 28,386 127  2,00 1911,31 30,072 120  3,00 1913,48 38,219 120  4,00 1914,73 21,120 125  Gesamt 1912,90 29,906 492  Gesamt 1,00 1919,26 25,384 248  2,00 1919,71 25,468 248  3,00 1920,17 30,669 248  4,00 1920,09 20,998 248  Gesamt 1919,81 25,821 992 | 1492  (*SD*=132; *N*=127)    Abhängige Variable:anker_einstein_estimate  anker_version E_Quartiles Mittelwert Std.-Abweichung N  A 1,00 1926,83 19,172 121  2,00 1927,59 16,878 128  3,00 1926,44 19,463 128  4,00 1925,54 19,492 123  Gesamt 1926,60 18,728 500  B 1,00 1912,06 28,386 127  2,00 1911,31 30,072 120  3,00 1913,48 38,219 120  4,00 1914,73 21,120 125  Gesamt 1912,90 29,906 492  Gesamt 1,00 1919,26 25,384 248  2,00 1919,71 25,468 248  3,00 1920,17 30,669 248  4,00 1920,09 20,998 248  Gesamt 1919,81 25,821 992  Deskriptive Statistiken  Abhängige Variable:anker_einstein_estimate  anker_version E_Quartiles Mittelwert Std.-Abweichung N  A 1,00 1926,83 19,172 121  2,00 1927,59 16,878 128  3,00 1926,44 19,463 128  4,00 1925,54 19,492 123  Gesamt 1926,60 18,728 500  B 1,00 1912,06 28,386 127  2,00 1911,31 30,072 120  3,00 1913,48 38,219 120  4,00 1914,73 21,120 125  Gesamt 1912,90 29,906 492  Gesamt 1,00 1919,26 25,384 248  2,00 1919,71 25,468 248  3,00 1920,17 30,669 248  4,00 1920,09 20,998 248  Gesamt 1919,81 25,821 992 | 1486  (*SD*=142; *N*=127)    Abhängige Variable:anker_einstein_estimate  anker_version E_Quartiles Mittelwert Std.-Abweichung N  A 1,00 1926,83 19,172 121  2,00 1927,59 16,878 128  3,00 1926,44 19,463 128  4,00 1925,54 19,492 123  Gesamt 1926,60 18,728 500  B 1,00 1912,06 28,386 127  2,00 1911,31 30,072 120  3,00 1913,48 38,219 120  4,00 1914,73 21,120 125  Gesamt 1912,90 29,906 492  Gesamt 1,00 1919,26 25,384 248  2,00 1919,71 25,468 248  3,00 1920,17 30,669 248  4,00 1920,09 20,998 248  Gesamt 1919,81 25,821 992  Deskriptive Statistiken  Abhängige Variable:anker_einstein_estimate  anker_version E_Quartiles Mittelwert Std.-Abweichung N  A 1,00 1926,83 19,172 121  2,00 1927,59 16,878 128  3,00 1926,44 19,463 128  4,00 1925,54 19,492 123  Gesamt 1926,60 18,728 500  B 1,00 1912,06 28,386 127  2,00 1911,31 30,072 120  3,00 1913,48 38,219 120  4,00 1914,73 21,120 125  Gesamt 1912,90 29,906 492  Gesamt 1,00 1919,26 25,384 248  2,00 1919,71 25,468 248  3,00 1920,17 30,669 248  4,00 1920,09 20,998 248  Gesamt 1919,81 25,821 992 | 1477  (*SD*=144; *N*=113)    Abhängige Variable:anker_einstein_estimate  anker_version E_Quartiles Mittelwert Std.-Abweichung N  A 1,00 1926,83 19,172 121  2,00 1927,59 16,878 128  3,00 1926,44 19,463 128  4,00 1925,54 19,492 123  Gesamt 1926,60 18,728 500  B 1,00 1912,06 28,386 127  2,00 1911,31 30,072 120  3,00 1913,48 38,219 120  4,00 1914,73 21,120 125  Gesamt 1912,90 29,906 492  Gesamt 1,00 1919,26 25,384 248  2,00 1919,71 25,468 248  3,00 1920,17 30,669 248  4,00 1920,09 20,998 248  Gesamt 1919,81 25,821 992  Deskriptive Statistiken  Abhängige Variable:anker_einstein_estimate  anker_version E_Quartiles Mittelwert Std.-Abweichung N  A 1,00 1926,83 19,172 121  2,00 1927,59 16,878 128  3,00 1926,44 19,463 128  4,00 1925,54 19,492 123  Gesamt 1926,60 18,728 500  B 1,00 1912,06 28,386 127  2,00 1911,31 30,072 120  3,00 1913,48 38,219 120  4,00 1914,73 21,120 125  Gesamt 1912,90 29,906 492  Gesamt 1,00 1919,26 25,384 248  2,00 1919,71 25,468 248  3,00 1920,17 30,669 248  4,00 1920,09 20,998 248  Gesamt 1919,81 25,821 992 |
|  | High (A) | 1626  (*SD*=118; *N*=123)    Abhängige Variable:anker_einstein_estimate  anker_version E_Quartiles Mittelwert Std.-Abweichung N  A 1,00 1926,83 19,172 121  2,00 1927,59 16,878 128  3,00 1926,44 19,463 128  4,00 1925,54 19,492 123  Gesamt 1926,60 18,728 500  B 1,00 1912,06 28,386 127  2,00 1911,31 30,072 120  3,00 1913,48 38,219 120  4,00 1914,73 21,120 125  Gesamt 1912,90 29,906 492  Gesamt 1,00 1919,26 25,384 248  2,00 1919,71 25,468 248  3,00 1920,17 30,669 248  4,00 1920,09 20,998 248  Gesamt 1919,81 25,821 992  Deskriptive Statistiken  Abhängige Variable:anker_einstein_estimate  anker_version E_Quartiles Mittelwert Std.-Abweichung N  A 1,00 1926,83 19,172 121  2,00 1927,59 16,878 128  3,00 1926,44 19,463 128  4,00 1925,54 19,492 123  Gesamt 1926,60 18,728 500  B 1,00 1912,06 28,386 127  2,00 1911,31 30,072 120  3,00 1913,48 38,219 120  4,00 1914,73 21,120 125  Gesamt 1912,90 29,906 492  Gesamt 1,00 1919,26 25,384 248  2,00 1919,71 25,468 248  3,00 1920,17 30,669 248  4,00 1920,09 20,998 248  Gesamt 1919,81 25,821 992 | 1626  (*SD*=118; *N*=121)    Abhängige Variable:anker_einstein_estimate  anker_version E_Quartiles Mittelwert Std.-Abweichung N  A 1,00 1926,83 19,172 121  2,00 1927,59 16,878 128  3,00 1926,44 19,463 128  4,00 1925,54 19,492 123  Gesamt 1926,60 18,728 500  B 1,00 1912,06 28,386 127  2,00 1911,31 30,072 120  3,00 1913,48 38,219 120  4,00 1914,73 21,120 125  Gesamt 1912,90 29,906 492  Gesamt 1,00 1919,26 25,384 248  2,00 1919,71 25,468 248  3,00 1920,17 30,669 248  4,00 1920,09 20,998 248  Gesamt 1919,81 25,821 992  Deskriptive Statistiken  Abhängige Variable:anker_einstein_estimate  anker_version E_Quartiles Mittelwert Std.-Abweichung N  A 1,00 1926,83 19,172 121  2,00 1927,59 16,878 128  3,00 1926,44 19,463 128  4,00 1925,54 19,492 123  Gesamt 1926,60 18,728 500  B 1,00 1912,06 28,386 127  2,00 1911,31 30,072 120  3,00 1913,48 38,219 120  4,00 1914,73 21,120 125  Gesamt 1912,90 29,906 492  Gesamt 1,00 1919,26 25,384 248  2,00 1919,71 25,468 248  3,00 1920,17 30,669 248  4,00 1920,09 20,998 248  Gesamt 1919,81 25,821 992 | 1614  (*SD*=103; *N*=121)    Abhängige Variable:anker_einstein_estimate  anker_version E_Quartiles Mittelwert Std.-Abweichung N  A 1,00 1926,83 19,172 121  2,00 1927,59 16,878 128  3,00 1926,44 19,463 128  4,00 1925,54 19,492 123  Gesamt 1926,60 18,728 500  B 1,00 1912,06 28,386 127  2,00 1911,31 30,072 120  3,00 1913,48 38,219 120  4,00 1914,73 21,120 125  Gesamt 1912,90 29,906 492  Gesamt 1,00 1919,26 25,384 248  2,00 1919,71 25,468 248  3,00 1920,17 30,669 248  4,00 1920,09 20,998 248  Gesamt 1919,81 25,821 992  Deskriptive Statistiken  Abhängige Variable:anker_einstein_estimate  anker_version E_Quartiles Mittelwert Std.-Abweichung N  A 1,00 1926,83 19,172 121  2,00 1927,59 16,878 128  3,00 1926,44 19,463 128  4,00 1925,54 19,492 123  Gesamt 1926,60 18,728 500  B 1,00 1912,06 28,386 127  2,00 1911,31 30,072 120  3,00 1913,48 38,219 120  4,00 1914,73 21,120 125  Gesamt 1912,90 29,906 492  Gesamt 1,00 1919,26 25,384 248  2,00 1919,71 25,468 248  3,00 1920,17 30,669 248  4,00 1920,09 20,998 248  Gesamt 1919,81 25,821 992 | 1600  (*SD*=122; *N*=135)    Abhängige Variable:anker_einstein_estimate  anker_version E_Quartiles Mittelwert Std.-Abweichung N  A 1,00 1926,83 19,172 121  2,00 1927,59 16,878 128  3,00 1926,44 19,463 128  4,00 1925,54 19,492 123  Gesamt 1926,60 18,728 500  B 1,00 1912,06 28,386 127  2,00 1911,31 30,072 120  3,00 1913,48 38,219 120  4,00 1914,73 21,120 125  Gesamt 1912,90 29,906 492  Gesamt 1,00 1919,26 25,384 248  2,00 1919,71 25,468 248  3,00 1920,17 30,669 248  4,00 1920,09 20,998 248  Gesamt 1919,81 25,821 992  Deskriptive Statistiken  Abhängige Variable:anker_einstein_estimate  anker_version E_Quartiles Mittelwert Std.-Abweichung N  A 1,00 1926,83 19,172 121  2,00 1927,59 16,878 128  3,00 1926,44 19,463 128  4,00 1925,54 19,492 123  Gesamt 1926,60 18,728 500  B 1,00 1912,06 28,386 127  2,00 1911,31 30,072 120  3,00 1913,48 38,219 120  4,00 1914,73 21,120 125  Gesamt 1912,90 29,906 492  Gesamt 1,00 1919,26 25,384 248  2,00 1919,71 25,468 248  3,00 1920,17 30,669 248  4,00 1920,09 20,998 248  Gesamt 1919,81 25,821 992 |
| Question Gandhi | Low (A) | 70  (*SD*=11; *N*=123)    Abhängige Variable:anker_einstein_estimate  anker_version E_Quartiles Mittelwert Std.-Abweichung N  A 1,00 1926,83 19,172 121  2,00 1927,59 16,878 128  3,00 1926,44 19,463 128  4,00 1925,54 19,492 123  Gesamt 1926,60 18,728 500  B 1,00 1912,06 28,386 127  2,00 1911,31 30,072 120  3,00 1913,48 38,219 120  4,00 1914,73 21,120 125  Gesamt 1912,90 29,906 492  Gesamt 1,00 1919,26 25,384 248  2,00 1919,71 25,468 248  3,00 1920,17 30,669 248  4,00 1920,09 20,998 248  Gesamt 1919,81 25,821 992  Deskriptive Statistiken  Abhängige Variable:anker_einstein_estimate  anker_version E_Quartiles Mittelwert Std.-Abweichung N  A 1,00 1926,83 19,172 121  2,00 1927,59 16,878 128  3,00 1926,44 19,463 128  4,00 1925,54 19,492 123  Gesamt 1926,60 18,728 500  B 1,00 1912,06 28,386 127  2,00 1911,31 30,072 120  3,00 1913,48 38,219 120  4,00 1914,73 21,120 125  Gesamt 1912,90 29,906 492  Gesamt 1,00 1919,26 25,384 248  2,00 1919,71 25,468 248  3,00 1920,17 30,669 248  4,00 1920,09 20,998 248  Gesamt 1919,81 25,821 992 | 70  (*SD*=10; *N*=121)    Abhängige Variable:anker_einstein_estimate  anker_version E_Quartiles Mittelwert Std.-Abweichung N  A 1,00 1926,83 19,172 121  2,00 1927,59 16,878 128  3,00 1926,44 19,463 128  4,00 1925,54 19,492 123  Gesamt 1926,60 18,728 500  B 1,00 1912,06 28,386 127  2,00 1911,31 30,072 120  3,00 1913,48 38,219 120  4,00 1914,73 21,120 125  Gesamt 1912,90 29,906 492  Gesamt 1,00 1919,26 25,384 248  2,00 1919,71 25,468 248  3,00 1920,17 30,669 248  4,00 1920,09 20,998 248  Gesamt 1919,81 25,821 992  Deskriptive Statistiken  Abhängige Variable:anker_einstein_estimate  anker_version E_Quartiles Mittelwert Std.-Abweichung N  A 1,00 1926,83 19,172 121  2,00 1927,59 16,878 128  3,00 1926,44 19,463 128  4,00 1925,54 19,492 123  Gesamt 1926,60 18,728 500  B 1,00 1912,06 28,386 127  2,00 1911,31 30,072 120  3,00 1913,48 38,219 120  4,00 1914,73 21,120 125  Gesamt 1912,90 29,906 492  Gesamt 1,00 1919,26 25,384 248  2,00 1919,71 25,468 248  3,00 1920,17 30,669 248  4,00 1920,09 20,998 248  Gesamt 1919,81 25,821 992 | 72  (*SD*=10; *N*=121)    Abhängige Variable:anker_einstein_estimate  anker_version E_Quartiles Mittelwert Std.-Abweichung N  A 1,00 1926,83 19,172 121  2,00 1927,59 16,878 128  3,00 1926,44 19,463 128  4,00 1925,54 19,492 123  Gesamt 1926,60 18,728 500  B 1,00 1912,06 28,386 127  2,00 1911,31 30,072 120  3,00 1913,48 38,219 120  4,00 1914,73 21,120 125  Gesamt 1912,90 29,906 492  Gesamt 1,00 1919,26 25,384 248  2,00 1919,71 25,468 248  3,00 1920,17 30,669 248  4,00 1920,09 20,998 248  Gesamt 1919,81 25,821 992  Deskriptive Statistiken  Abhängige Variable:anker_einstein_estimate  anker_version E_Quartiles Mittelwert Std.-Abweichung N  A 1,00 1926,83 19,172 121  2,00 1927,59 16,878 128  3,00 1926,44 19,463 128  4,00 1925,54 19,492 123  Gesamt 1926,60 18,728 500  B 1,00 1912,06 28,386 127  2,00 1911,31 30,072 120  3,00 1913,48 38,219 120  4,00 1914,73 21,120 125  Gesamt 1912,90 29,906 492  Gesamt 1,00 1919,26 25,384 248  2,00 1919,71 25,468 248  3,00 1920,17 30,669 248  4,00 1920,09 20,998 248  Gesamt 1919,81 25,821 992 | 70  (*SD*=10; *N*=135)    Abhängige Variable:anker_einstein_estimate  anker_version E_Quartiles Mittelwert Std.-Abweichung N  A 1,00 1926,83 19,172 121  2,00 1927,59 16,878 128  3,00 1926,44 19,463 128  4,00 1925,54 19,492 123  Gesamt 1926,60 18,728 500  B 1,00 1912,06 28,386 127  2,00 1911,31 30,072 120  3,00 1913,48 38,219 120  4,00 1914,73 21,120 125  Gesamt 1912,90 29,906 492  Gesamt 1,00 1919,26 25,384 248  2,00 1919,71 25,468 248  3,00 1920,17 30,669 248  4,00 1920,09 20,998 248  Gesamt 1919,81 25,821 992  Deskriptive Statistiken  Abhängige Variable:anker_einstein_estimate  anker_version E_Quartiles Mittelwert Std.-Abweichung N  A 1,00 1926,83 19,172 121  2,00 1927,59 16,878 128  3,00 1926,44 19,463 128  4,00 1925,54 19,492 123  Gesamt 1926,60 18,728 500  B 1,00 1912,06 28,386 127  2,00 1911,31 30,072 120  3,00 1913,48 38,219 120  4,00 1914,73 21,120 125  Gesamt 1912,90 29,906 492  Gesamt 1,00 1919,26 25,384 248  2,00 1919,71 25,468 248  3,00 1920,17 30,669 248  4,00 1920,09 20,998 248  Gesamt 1919,81 25,821 992 |
|  | High (B) | 80  (*SD*=9; *N*=125)    Abhängige Variable:anker_einstein_estimate  anker_version E_Quartiles Mittelwert Std.-Abweichung N  A 1,00 1926,83 19,172 121  2,00 1927,59 16,878 128  3,00 1926,44 19,463 128  4,00 1925,54 19,492 123  Gesamt 1926,60 18,728 500  B 1,00 1912,06 28,386 127  2,00 1911,31 30,072 120  3,00 1913,48 38,219 120  4,00 1914,73 21,120 125  Gesamt 1912,90 29,906 492  Gesamt 1,00 1919,26 25,384 248  2,00 1919,71 25,468 248  3,00 1920,17 30,669 248  4,00 1920,09 20,998 248  Gesamt 1919,81 25,821 992  Deskriptive Statistiken  Abhängige Variable:anker_einstein_estimate  anker_version E_Quartiles Mittelwert Std.-Abweichung N  A 1,00 1926,83 19,172 121  2,00 1927,59 16,878 128  3,00 1926,44 19,463 128  4,00 1925,54 19,492 123  Gesamt 1926,60 18,728 500  B 1,00 1912,06 28,386 127  2,00 1911,31 30,072 120  3,00 1913,48 38,219 120  4,00 1914,73 21,120 125  Gesamt 1912,90 29,906 492  Gesamt 1,00 1919,26 25,384 248  2,00 1919,71 25,468 248  3,00 1920,17 30,669 248  4,00 1920,09 20,998 248  Gesamt 1919,81 25,821 992 | 79  (*SD*=9; *N*=127)    Abhängige Variable:anker_einstein_estimate  anker_version E_Quartiles Mittelwert Std.-Abweichung N  A 1,00 1926,83 19,172 121  2,00 1927,59 16,878 128  3,00 1926,44 19,463 128  4,00 1925,54 19,492 123  Gesamt 1926,60 18,728 500  B 1,00 1912,06 28,386 127  2,00 1911,31 30,072 120  3,00 1913,48 38,219 120  4,00 1914,73 21,120 125  Gesamt 1912,90 29,906 492  Gesamt 1,00 1919,26 25,384 248  2,00 1919,71 25,468 248  3,00 1920,17 30,669 248  4,00 1920,09 20,998 248  Gesamt 1919,81 25,821 992  Deskriptive Statistiken  Abhängige Variable:anker_einstein_estimate  anker_version E_Quartiles Mittelwert Std.-Abweichung N  A 1,00 1926,83 19,172 121  2,00 1927,59 16,878 128  3,00 1926,44 19,463 128  4,00 1925,54 19,492 123  Gesamt 1926,60 18,728 500  B 1,00 1912,06 28,386 127  2,00 1911,31 30,072 120  3,00 1913,48 38,219 120  4,00 1914,73 21,120 125  Gesamt 1912,90 29,906 492  Gesamt 1,00 1919,26 25,384 248  2,00 1919,71 25,468 248  3,00 1920,17 30,669 248  4,00 1920,09 20,998 248  Gesamt 1919,81 25,821 992 | 80  (*SD*=8; *N*=127)    Abhängige Variable:anker_einstein_estimate  anker_version E_Quartiles Mittelwert Std.-Abweichung N  A 1,00 1926,83 19,172 121  2,00 1927,59 16,878 128  3,00 1926,44 19,463 128  4,00 1925,54 19,492 123  Gesamt 1926,60 18,728 500  B 1,00 1912,06 28,386 127  2,00 1911,31 30,072 120  3,00 1913,48 38,219 120  4,00 1914,73 21,120 125  Gesamt 1912,90 29,906 492  Gesamt 1,00 1919,26 25,384 248  2,00 1919,71 25,468 248  3,00 1920,17 30,669 248  4,00 1920,09 20,998 248  Gesamt 1919,81 25,821 992  Deskriptive Statistiken  Abhängige Variable:anker_einstein_estimate  anker_version E_Quartiles Mittelwert Std.-Abweichung N  A 1,00 1926,83 19,172 121  2,00 1927,59 16,878 128  3,00 1926,44 19,463 128  4,00 1925,54 19,492 123  Gesamt 1926,60 18,728 500  B 1,00 1912,06 28,386 127  2,00 1911,31 30,072 120  3,00 1913,48 38,219 120  4,00 1914,73 21,120 125  Gesamt 1912,90 29,906 492  Gesamt 1,00 1919,26 25,384 248  2,00 1919,71 25,468 248  3,00 1920,17 30,669 248  4,00 1920,09 20,998 248  Gesamt 1919,81 25,821 992 | 77  (*SD*=9; *N*=113)    Abhängige Variable:anker_einstein_estimate  anker_version E_Quartiles Mittelwert Std.-Abweichung N  A 1,00 1926,83 19,172 121  2,00 1927,59 16,878 128  3,00 1926,44 19,463 128  4,00 1925,54 19,492 123  Gesamt 1926,60 18,728 500  B 1,00 1912,06 28,386 127  2,00 1911,31 30,072 120  3,00 1913,48 38,219 120  4,00 1914,73 21,120 125  Gesamt 1912,90 29,906 492  Gesamt 1,00 1919,26 25,384 248  2,00 1919,71 25,468 248  3,00 1920,17 30,669 248  4,00 1920,09 20,998 248  Gesamt 1919,81 25,821 992  Deskriptive Statistiken  Abhängige Variable:anker_einstein_estimate  anker_version E_Quartiles Mittelwert Std.-Abweichung N  A 1,00 1926,83 19,172 121  2,00 1927,59 16,878 128  3,00 1926,44 19,463 128  4,00 1925,54 19,492 123  Gesamt 1926,60 18,728 500  B 1,00 1912,06 28,386 127  2,00 1911,31 30,072 120  3,00 1913,48 38,219 120  4,00 1914,73 21,120 125  Gesamt 1912,90 29,906 492  Gesamt 1,00 1919,26 25,384 248  2,00 1919,71 25,468 248  3,00 1920,17 30,669 248  4,00 1920,09 20,998 248  Gesamt 1919,81 25,821 992 |
| Question Sahara | Low (A) | 81  (*SD*=118; *N*=123)    Abhängige Variable:anker_einstein_estimate  anker_version E_Quartiles Mittelwert Std.-Abweichung N  A 1,00 1926,83 19,172 121  2,00 1927,59 16,878 128  3,00 1926,44 19,463 128  4,00 1925,54 19,492 123  Gesamt 1926,60 18,728 500  B 1,00 1912,06 28,386 127  2,00 1911,31 30,072 120  3,00 1913,48 38,219 120  4,00 1914,73 21,120 125  Gesamt 1912,90 29,906 492  Gesamt 1,00 1919,26 25,384 248  2,00 1919,71 25,468 248  3,00 1920,17 30,669 248  4,00 1920,09 20,998 248  Gesamt 1919,81 25,821 992  Deskriptive Statistiken  Abhängige Variable:anker_einstein_estimate  anker_version E_Quartiles Mittelwert Std.-Abweichung N  A 1,00 1926,83 19,172 121  2,00 1927,59 16,878 128  3,00 1926,44 19,463 128  4,00 1925,54 19,492 123  Gesamt 1926,60 18,728 500  B 1,00 1912,06 28,386 127  2,00 1911,31 30,072 120  3,00 1913,48 38,219 120  4,00 1914,73 21,120 125  Gesamt 1912,90 29,906 492  Gesamt 1,00 1919,26 25,384 248  2,00 1919,71 25,468 248  3,00 1920,17 30,669 248  4,00 1920,09 20,998 248  Gesamt 1919,81 25,821 992 | 71  (*SD*=118; *N*=121)    Abhängige Variable:anker_einstein_estimate  anker_version E_Quartiles Mittelwert Std.-Abweichung N  A 1,00 1926,83 19,172 121  2,00 1927,59 16,878 128  3,00 1926,44 19,463 128  4,00 1925,54 19,492 123  Gesamt 1926,60 18,728 500  B 1,00 1912,06 28,386 127  2,00 1911,31 30,072 120  3,00 1913,48 38,219 120  4,00 1914,73 21,120 125  Gesamt 1912,90 29,906 492  Gesamt 1,00 1919,26 25,384 248  2,00 1919,71 25,468 248  3,00 1920,17 30,669 248  4,00 1920,09 20,998 248  Gesamt 1919,81 25,821 992  Deskriptive Statistiken  Abhängige Variable:anker_einstein_estimate  anker_version E_Quartiles Mittelwert Std.-Abweichung N  A 1,00 1926,83 19,172 121  2,00 1927,59 16,878 128  3,00 1926,44 19,463 128  4,00 1925,54 19,492 123  Gesamt 1926,60 18,728 500  B 1,00 1912,06 28,386 127  2,00 1911,31 30,072 120  3,00 1913,48 38,219 120  4,00 1914,73 21,120 125  Gesamt 1912,90 29,906 492  Gesamt 1,00 1919,26 25,384 248  2,00 1919,71 25,468 248  3,00 1920,17 30,669 248  4,00 1920,09 20,998 248  Gesamt 1919,81 25,821 992 | 66  (*SD*=103; *N*=121)    Abhängige Variable:anker_einstein_estimate  anker_version E_Quartiles Mittelwert Std.-Abweichung N  A 1,00 1926,83 19,172 121  2,00 1927,59 16,878 128  3,00 1926,44 19,463 128  4,00 1925,54 19,492 123  Gesamt 1926,60 18,728 500  B 1,00 1912,06 28,386 127  2,00 1911,31 30,072 120  3,00 1913,48 38,219 120  4,00 1914,73 21,120 125  Gesamt 1912,90 29,906 492  Gesamt 1,00 1919,26 25,384 248  2,00 1919,71 25,468 248  3,00 1920,17 30,669 248  4,00 1920,09 20,998 248  Gesamt 1919,81 25,821 992  Deskriptive Statistiken  Abhängige Variable:anker_einstein_estimate  anker_version E_Quartiles Mittelwert Std.-Abweichung N  A 1,00 1926,83 19,172 121  2,00 1927,59 16,878 128  3,00 1926,44 19,463 128  4,00 1925,54 19,492 123  Gesamt 1926,60 18,728 500  B 1,00 1912,06 28,386 127  2,00 1911,31 30,072 120  3,00 1913,48 38,219 120  4,00 1914,73 21,120 125  Gesamt 1912,90 29,906 492  Gesamt 1,00 1919,26 25,384 248  2,00 1919,71 25,468 248  3,00 1920,17 30,669 248  4,00 1920,09 20,998 248  Gesamt 1919,81 25,821 992 | 105  (*SD*=122; *N*=135)    Abhängige Variable:anker_einstein_estimate  anker_version E_Quartiles Mittelwert Std.-Abweichung N  A 1,00 1926,83 19,172 121  2,00 1927,59 16,878 128  3,00 1926,44 19,463 128  4,00 1925,54 19,492 123  Gesamt 1926,60 18,728 500  B 1,00 1912,06 28,386 127  2,00 1911,31 30,072 120  3,00 1913,48 38,219 120  4,00 1914,73 21,120 125  Gesamt 1912,90 29,906 492  Gesamt 1,00 1919,26 25,384 248  2,00 1919,71 25,468 248  3,00 1920,17 30,669 248  4,00 1920,09 20,998 248  Gesamt 1919,81 25,821 992  Deskriptive Statistiken  Abhängige Variable:anker_einstein_estimate  anker_version E_Quartiles Mittelwert Std.-Abweichung N  A 1,00 1926,83 19,172 121  2,00 1927,59 16,878 128  3,00 1926,44 19,463 128  4,00 1925,54 19,492 123  Gesamt 1926,60 18,728 500  B 1,00 1912,06 28,386 127  2,00 1911,31 30,072 120  3,00 1913,48 38,219 120  4,00 1914,73 21,120 125  Gesamt 1912,90 29,906 492  Gesamt 1,00 1919,26 25,384 248  2,00 1919,71 25,468 248  3,00 1920,17 30,669 248  4,00 1920,09 20,998 248  Gesamt 1919,81 25,821 992 |
|  | High (B) | 127  (*SD*=169; *N*=125)    Abhängige Variable:anker_einstein_estimate  anker_version E_Quartiles Mittelwert Std.-Abweichung N  A 1,00 1926,83 19,172 121  2,00 1927,59 16,878 128  3,00 1926,44 19,463 128  4,00 1925,54 19,492 123  Gesamt 1926,60 18,728 500  B 1,00 1912,06 28,386 127  2,00 1911,31 30,072 120  3,00 1913,48 38,219 120  4,00 1914,73 21,120 125  Gesamt 1912,90 29,906 492  Gesamt 1,00 1919,26 25,384 248  2,00 1919,71 25,468 248  3,00 1920,17 30,669 248  4,00 1920,09 20,998 248  Gesamt 1919,81 25,821 992  Deskriptive Statistiken  Abhängige Variable:anker_einstein_estimate  anker_version E_Quartiles Mittelwert Std.-Abweichung N  A 1,00 1926,83 19,172 121  2,00 1927,59 16,878 128  3,00 1926,44 19,463 128  4,00 1925,54 19,492 123  Gesamt 1926,60 18,728 500  B 1,00 1912,06 28,386 127  2,00 1911,31 30,072 120  3,00 1913,48 38,219 120  4,00 1914,73 21,120 125  Gesamt 1912,90 29,906 492  Gesamt 1,00 1919,26 25,384 248  2,00 1919,71 25,468 248  3,00 1920,17 30,669 248  4,00 1920,09 20,998 248  Gesamt 1919,81 25,821 992 | 124  (*SD*=155; *N*=127)    Abhängige Variable:anker_einstein_estimate  anker_version E_Quartiles Mittelwert Std.-Abweichung N  A 1,00 1926,83 19,172 121  2,00 1927,59 16,878 128  3,00 1926,44 19,463 128  4,00 1925,54 19,492 123  Gesamt 1926,60 18,728 500  B 1,00 1912,06 28,386 127  2,00 1911,31 30,072 120  3,00 1913,48 38,219 120  4,00 1914,73 21,120 125  Gesamt 1912,90 29,906 492  Gesamt 1,00 1919,26 25,384 248  2,00 1919,71 25,468 248  3,00 1920,17 30,669 248  4,00 1920,09 20,998 248  Gesamt 1919,81 25,821 992  Deskriptive Statistiken  Abhängige Variable:anker_einstein_estimate  anker_version E_Quartiles Mittelwert Std.-Abweichung N  A 1,00 1926,83 19,172 121  2,00 1927,59 16,878 128  3,00 1926,44 19,463 128  4,00 1925,54 19,492 123  Gesamt 1926,60 18,728 500  B 1,00 1912,06 28,386 127  2,00 1911,31 30,072 120  3,00 1913,48 38,219 120  4,00 1914,73 21,120 125  Gesamt 1912,90 29,906 492  Gesamt 1,00 1919,26 25,384 248  2,00 1919,71 25,468 248  3,00 1920,17 30,669 248  4,00 1920,09 20,998 248  Gesamt 1919,81 25,821 992 | 156  (*SD*=226; *N*=127)    Abhängige Variable:anker_einstein_estimate  anker_version E_Quartiles Mittelwert Std.-Abweichung N  A 1,00 1926,83 19,172 121  2,00 1927,59 16,878 128  3,00 1926,44 19,463 128  4,00 1925,54 19,492 123  Gesamt 1926,60 18,728 500  B 1,00 1912,06 28,386 127  2,00 1911,31 30,072 120  3,00 1913,48 38,219 120  4,00 1914,73 21,120 125  Gesamt 1912,90 29,906 492  Gesamt 1,00 1919,26 25,384 248  2,00 1919,71 25,468 248  3,00 1920,17 30,669 248  4,00 1920,09 20,998 248  Gesamt 1919,81 25,821 992  Deskriptive Statistiken  Abhängige Variable:anker_einstein_estimate  anker_version E_Quartiles Mittelwert Std.-Abweichung N  A 1,00 1926,83 19,172 121  2,00 1927,59 16,878 128  3,00 1926,44 19,463 128  4,00 1925,54 19,492 123  Gesamt 1926,60 18,728 500  B 1,00 1912,06 28,386 127  2,00 1911,31 30,072 120  3,00 1913,48 38,219 120  4,00 1914,73 21,120 125  Gesamt 1912,90 29,906 492  Gesamt 1,00 1919,26 25,384 248  2,00 1919,71 25,468 248  3,00 1920,17 30,669 248  4,00 1920,09 20,998 248  Gesamt 1919,81 25,821 992 | 108  (*SD*=119; *N*=113)    Abhängige Variable:anker_einstein_estimate  anker_version E_Quartiles Mittelwert Std.-Abweichung N  A 1,00 1926,83 19,172 121  2,00 1927,59 16,878 128  3,00 1926,44 19,463 128  4,00 1925,54 19,492 123  Gesamt 1926,60 18,728 500  B 1,00 1912,06 28,386 127  2,00 1911,31 30,072 120  3,00 1913,48 38,219 120  4,00 1914,73 21,120 125  Gesamt 1912,90 29,906 492  Gesamt 1,00 1919,26 25,384 248  2,00 1919,71 25,468 248  3,00 1920,17 30,669 248  4,00 1920,09 20,998 248  Gesamt 1919,81 25,821 992  Deskriptive Statistiken  Abhängige Variable:anker_einstein_estimate  anker_version E_Quartiles Mittelwert Std.-Abweichung N  A 1,00 1926,83 19,172 121  2,00 1927,59 16,878 128  3,00 1926,44 19,463 128  4,00 1925,54 19,492 123  Gesamt 1926,60 18,728 500  B 1,00 1912,06 28,386 127  2,00 1911,31 30,072 120  3,00 1913,48 38,219 120  4,00 1914,73 21,120 125  Gesamt 1912,90 29,906 492  Gesamt 1,00 1919,26 25,384 248  2,00 1919,71 25,468 248  3,00 1920,17 30,669 248  4,00 1920,09 20,998 248  Gesamt 1919,81 25,821 992 |
| **Agreeableness** | | **Q1 (0-25%)** | **Q2 (25-50%)** | **Q3 (50-75%)** | **Q 4 (75-100%)** |
| Question Einstein | Low (B) | 1910  (*SD*=47; *N*=122)    Abhängige Variable:anker_einstein_estimate  anker_version E_Quartiles Mittelwert Std.-Abweichung N  A 1,00 1926,83 19,172 121  2,00 1927,59 16,878 128  3,00 1926,44 19,463 128  4,00 1925,54 19,492 123  Gesamt 1926,60 18,728 500  B 1,00 1912,06 28,386 127  2,00 1911,31 30,072 120  3,00 1913,48 38,219 120  4,00 1914,73 21,120 125  Gesamt 1912,90 29,906 492  Gesamt 1,00 1919,26 25,384 248  2,00 1919,71 25,468 248  3,00 1920,17 30,669 248  4,00 1920,09 20,998 248  Gesamt 1919,81 25,821 992  Deskriptive Statistiken  Abhängige Variable:anker_einstein_estimate  anker_version E_Quartiles Mittelwert Std.-Abweichung N  A 1,00 1926,83 19,172 121  2,00 1927,59 16,878 128  3,00 1926,44 19,463 128  4,00 1925,54 19,492 123  Gesamt 1926,60 18,728 500  B 1,00 1912,06 28,386 127  2,00 1911,31 30,072 120  3,00 1913,48 38,219 120  4,00 1914,73 21,120 125  Gesamt 1912,90 29,906 492  Gesamt 1,00 1919,26 25,384 248  2,00 1919,71 25,468 248  3,00 1920,17 30,669 248  4,00 1920,09 20,998 248  Gesamt 1919,81 25,821 992 | 1918  (*SD*=17; *N*=124)    Abhängige Variable:anker_einstein_estimate  anker_version E_Quartiles Mittelwert Std.-Abweichung N  A 1,00 1926,83 19,172 121  2,00 1927,59 16,878 128  3,00 1926,44 19,463 128  4,00 1925,54 19,492 123  Gesamt 1926,60 18,728 500  B 1,00 1912,06 28,386 127  2,00 1911,31 30,072 120  3,00 1913,48 38,219 120  4,00 1914,73 21,120 125  Gesamt 1912,90 29,906 492  Gesamt 1,00 1919,26 25,384 248  2,00 1919,71 25,468 248  3,00 1920,17 30,669 248  4,00 1920,09 20,998 248  Gesamt 1919,81 25,821 992  Deskriptive Statistiken  Abhängige Variable:anker_einstein_estimate  anker_version E_Quartiles Mittelwert Std.-Abweichung N  A 1,00 1926,83 19,172 121  2,00 1927,59 16,878 128  3,00 1926,44 19,463 128  4,00 1925,54 19,492 123  Gesamt 1926,60 18,728 500  B 1,00 1912,06 28,386 127  2,00 1911,31 30,072 120  3,00 1913,48 38,219 120  4,00 1914,73 21,120 125  Gesamt 1912,90 29,906 492  Gesamt 1,00 1919,26 25,384 248  2,00 1919,71 25,468 248  3,00 1920,17 30,669 248  4,00 1920,09 20,998 248  Gesamt 1919,81 25,821 992 | 1914  (*SD*=23; *N*=122)    Abhängige Variable:anker_einstein_estimate  anker_version E_Quartiles Mittelwert Std.-Abweichung N  A 1,00 1926,83 19,172 121  2,00 1927,59 16,878 128  3,00 1926,44 19,463 128  4,00 1925,54 19,492 123  Gesamt 1926,60 18,728 500  B 1,00 1912,06 28,386 127  2,00 1911,31 30,072 120  3,00 1913,48 38,219 120  4,00 1914,73 21,120 125  Gesamt 1912,90 29,906 492  Gesamt 1,00 1919,26 25,384 248  2,00 1919,71 25,468 248  3,00 1920,17 30,669 248  4,00 1920,09 20,998 248  Gesamt 1919,81 25,821 992  Deskriptive Statistiken  Abhängige Variable:anker_einstein_estimate  anker_version E_Quartiles Mittelwert Std.-Abweichung N  A 1,00 1926,83 19,172 121  2,00 1927,59 16,878 128  3,00 1926,44 19,463 128  4,00 1925,54 19,492 123  Gesamt 1926,60 18,728 500  B 1,00 1912,06 28,386 127  2,00 1911,31 30,072 120  3,00 1913,48 38,219 120  4,00 1914,73 21,120 125  Gesamt 1912,90 29,906 492  Gesamt 1,00 1919,26 25,384 248  2,00 1919,71 25,468 248  3,00 1920,17 30,669 248  4,00 1920,09 20,998 248  Gesamt 1919,81 25,821 992 | 1909  (*SD*=23; *N*=124)    Abhängige Variable:anker_einstein_estimate  anker_version E_Quartiles Mittelwert Std.-Abweichung N  A 1,00 1926,83 19,172 121  2,00 1927,59 16,878 128  3,00 1926,44 19,463 128  4,00 1925,54 19,492 123  Gesamt 1926,60 18,728 500  B 1,00 1912,06 28,386 127  2,00 1911,31 30,072 120  3,00 1913,48 38,219 120  4,00 1914,73 21,120 125  Gesamt 1912,90 29,906 492  Gesamt 1,00 1919,26 25,384 248  2,00 1919,71 25,468 248  3,00 1920,17 30,669 248  4,00 1920,09 20,998 248  Gesamt 1919,81 25,821 992  Deskriptive Statistiken  Abhängige Variable:anker_einstein_estimate  anker_version E_Quartiles Mittelwert Std.-Abweichung N  A 1,00 1926,83 19,172 121  2,00 1927,59 16,878 128  3,00 1926,44 19,463 128  4,00 1925,54 19,492 123  Gesamt 1926,60 18,728 500  B 1,00 1912,06 28,386 127  2,00 1911,31 30,072 120  3,00 1913,48 38,219 120  4,00 1914,73 21,120 125  Gesamt 1912,90 29,906 492  Gesamt 1,00 1919,26 25,384 248  2,00 1919,71 25,468 248  3,00 1920,17 30,669 248  4,00 1920,09 20,998 248  Gesamt 1919,81 25,821 992 |
|  | High(A) | 1929  (*SD*=21; *N*=126)    Abhängige Variable:anker_einstein_estimate  anker_version E_Quartiles Mittelwert Std.-Abweichung N  A 1,00 1926,83 19,172 121  2,00 1927,59 16,878 128  3,00 1926,44 19,463 128  4,00 1925,54 19,492 123  Gesamt 1926,60 18,728 500  B 1,00 1912,06 28,386 127  2,00 1911,31 30,072 120  3,00 1913,48 38,219 120  4,00 1914,73 21,120 125  Gesamt 1912,90 29,906 492  Gesamt 1,00 1919,26 25,384 248  2,00 1919,71 25,468 248  3,00 1920,17 30,669 248  4,00 1920,09 20,998 248  Gesamt 1919,81 25,821 992  Deskriptive Statistiken  Abhängige Variable:anker_einstein_estimate  anker_version E_Quartiles Mittelwert Std.-Abweichung N  A 1,00 1926,83 19,172 121  2,00 1927,59 16,878 128  3,00 1926,44 19,463 128  4,00 1925,54 19,492 123  Gesamt 1926,60 18,728 500  B 1,00 1912,06 28,386 127  2,00 1911,31 30,072 120  3,00 1913,48 38,219 120  4,00 1914,73 21,120 125  Gesamt 1912,90 29,906 492  Gesamt 1,00 1919,26 25,384 248  2,00 1919,71 25,468 248  3,00 1920,17 30,669 248  4,00 1920,09 20,998 248  Gesamt 1919,81 25,821 992 | 1927  (*SD*=17; *N*=124)    Abhängige Variable:anker_einstein_estimate  anker_version E_Quartiles Mittelwert Std.-Abweichung N  A 1,00 1926,83 19,172 121  2,00 1927,59 16,878 128  3,00 1926,44 19,463 128  4,00 1925,54 19,492 123  Gesamt 1926,60 18,728 500  B 1,00 1912,06 28,386 127  2,00 1911,31 30,072 120  3,00 1913,48 38,219 120  4,00 1914,73 21,120 125  Gesamt 1912,90 29,906 492  Gesamt 1,00 1919,26 25,384 248  2,00 1919,71 25,468 248  3,00 1920,17 30,669 248  4,00 1920,09 20,998 248  Gesamt 1919,81 25,821 992  Deskriptive Statistiken  Abhängige Variable:anker_einstein_estimate  anker_version E_Quartiles Mittelwert Std.-Abweichung N  A 1,00 1926,83 19,172 121  2,00 1927,59 16,878 128  3,00 1926,44 19,463 128  4,00 1925,54 19,492 123  Gesamt 1926,60 18,728 500  B 1,00 1912,06 28,386 127  2,00 1911,31 30,072 120  3,00 1913,48 38,219 120  4,00 1914,73 21,120 125  Gesamt 1912,90 29,906 492  Gesamt 1,00 1919,26 25,384 248  2,00 1919,71 25,468 248  3,00 1920,17 30,669 248  4,00 1920,09 20,998 248  Gesamt 1919,81 25,821 992 | 1925  (*SD*=17; *N*=126)    Abhängige Variable:anker_einstein_estimate  anker_version E_Quartiles Mittelwert Std.-Abweichung N  A 1,00 1926,83 19,172 121  2,00 1927,59 16,878 128  3,00 1926,44 19,463 128  4,00 1925,54 19,492 123  Gesamt 1926,60 18,728 500  B 1,00 1912,06 28,386 127  2,00 1911,31 30,072 120  3,00 1913,48 38,219 120  4,00 1914,73 21,120 125  Gesamt 1912,90 29,906 492  Gesamt 1,00 1919,26 25,384 248  2,00 1919,71 25,468 248  3,00 1920,17 30,669 248  4,00 1920,09 20,998 248  Gesamt 1919,81 25,821 992  Deskriptive Statistiken  Abhängige Variable:anker_einstein_estimate  anker_version E_Quartiles Mittelwert Std.-Abweichung N  A 1,00 1926,83 19,172 121  2,00 1927,59 16,878 128  3,00 1926,44 19,463 128  4,00 1925,54 19,492 123  Gesamt 1926,60 18,728 500  B 1,00 1912,06 28,386 127  2,00 1911,31 30,072 120  3,00 1913,48 38,219 120  4,00 1914,73 21,120 125  Gesamt 1912,90 29,906 492  Gesamt 1,00 1919,26 25,384 248  2,00 1919,71 25,468 248  3,00 1920,17 30,669 248  4,00 1920,09 20,998 248  Gesamt 1919,81 25,821 992 | 1927  (*SD*=19; *N*=124)    Abhängige Variable:anker_einstein_estimate  anker_version E_Quartiles Mittelwert Std.-Abweichung N  A 1,00 1926,83 19,172 121  2,00 1927,59 16,878 128  3,00 1926,44 19,463 128  4,00 1925,54 19,492 123  Gesamt 1926,60 18,728 500  B 1,00 1912,06 28,386 127  2,00 1911,31 30,072 120  3,00 1913,48 38,219 120  4,00 1914,73 21,120 125  Gesamt 1912,90 29,906 492  Gesamt 1,00 1919,26 25,384 248  2,00 1919,71 25,468 248  3,00 1920,17 30,669 248  4,00 1920,09 20,998 248  Gesamt 1919,81 25,821 992  Deskriptive Statistiken  Abhängige Variable:anker_einstein_estimate  anker_version E_Quartiles Mittelwert Std.-Abweichung N  A 1,00 1926,83 19,172 121  2,00 1927,59 16,878 128  3,00 1926,44 19,463 128  4,00 1925,54 19,492 123  Gesamt 1926,60 18,728 500  B 1,00 1912,06 28,386 127  2,00 1911,31 30,072 120  3,00 1913,48 38,219 120  4,00 1914,73 21,120 125  Gesamt 1912,90 29,906 492  Gesamt 1,00 1919,26 25,384 248  2,00 1919,71 25,468 248  3,00 1920,17 30,669 248  4,00 1920,09 20,998 248  Gesamt 1919,81 25,821 992 |
| Question da Vinci | Low (B) | 1493  (*SD*=122; *N*=122)    Abhängige Variable:anker_einstein_estimate  anker_version E_Quartiles Mittelwert Std.-Abweichung N  A 1,00 1926,83 19,172 121  2,00 1927,59 16,878 128  3,00 1926,44 19,463 128  4,00 1925,54 19,492 123  Gesamt 1926,60 18,728 500  B 1,00 1912,06 28,386 127  2,00 1911,31 30,072 120  3,00 1913,48 38,219 120  4,00 1914,73 21,120 125  Gesamt 1912,90 29,906 492  Gesamt 1,00 1919,26 25,384 248  2,00 1919,71 25,468 248  3,00 1920,17 30,669 248  4,00 1920,09 20,998 248  Gesamt 1919,81 25,821 992  Deskriptive Statistiken  Abhängige Variable:anker_einstein_estimate  anker_version E_Quartiles Mittelwert Std.-Abweichung N  A 1,00 1926,83 19,172 121  2,00 1927,59 16,878 128  3,00 1926,44 19,463 128  4,00 1925,54 19,492 123  Gesamt 1926,60 18,728 500  B 1,00 1912,06 28,386 127  2,00 1911,31 30,072 120  3,00 1913,48 38,219 120  4,00 1914,73 21,120 125  Gesamt 1912,90 29,906 492  Gesamt 1,00 1919,26 25,384 248  2,00 1919,71 25,468 248  3,00 1920,17 30,669 248  4,00 1920,09 20,998 248  Gesamt 1919,81 25,821 992 | 1482  (*SD*=149; *N*=124)    Abhängige Variable:anker_einstein_estimate  anker_version E_Quartiles Mittelwert Std.-Abweichung N  A 1,00 1926,83 19,172 121  2,00 1927,59 16,878 128  3,00 1926,44 19,463 128  4,00 1925,54 19,492 123  Gesamt 1926,60 18,728 500  B 1,00 1912,06 28,386 127  2,00 1911,31 30,072 120  3,00 1913,48 38,219 120  4,00 1914,73 21,120 125  Gesamt 1912,90 29,906 492  Gesamt 1,00 1919,26 25,384 248  2,00 1919,71 25,468 248  3,00 1920,17 30,669 248  4,00 1920,09 20,998 248  Gesamt 1919,81 25,821 992  Deskriptive Statistiken  Abhängige Variable:anker_einstein_estimate  anker_version E_Quartiles Mittelwert Std.-Abweichung N  A 1,00 1926,83 19,172 121  2,00 1927,59 16,878 128  3,00 1926,44 19,463 128  4,00 1925,54 19,492 123  Gesamt 1926,60 18,728 500  B 1,00 1912,06 28,386 127  2,00 1911,31 30,072 120  3,00 1913,48 38,219 120  4,00 1914,73 21,120 125  Gesamt 1912,90 29,906 492  Gesamt 1,00 1919,26 25,384 248  2,00 1919,71 25,468 248  3,00 1920,17 30,669 248  4,00 1920,09 20,998 248  Gesamt 1919,81 25,821 992 | 1487  (*SD*=130; *N*=122)    Abhängige Variable:anker_einstein_estimate  anker_version E_Quartiles Mittelwert Std.-Abweichung N  A 1,00 1926,83 19,172 121  2,00 1927,59 16,878 128  3,00 1926,44 19,463 128  4,00 1925,54 19,492 123  Gesamt 1926,60 18,728 500  B 1,00 1912,06 28,386 127  2,00 1911,31 30,072 120  3,00 1913,48 38,219 120  4,00 1914,73 21,120 125  Gesamt 1912,90 29,906 492  Gesamt 1,00 1919,26 25,384 248  2,00 1919,71 25,468 248  3,00 1920,17 30,669 248  4,00 1920,09 20,998 248  Gesamt 1919,81 25,821 992  Deskriptive Statistiken  Abhängige Variable:anker_einstein_estimate  anker_version E_Quartiles Mittelwert Std.-Abweichung N  A 1,00 1926,83 19,172 121  2,00 1927,59 16,878 128  3,00 1926,44 19,463 128  4,00 1925,54 19,492 123  Gesamt 1926,60 18,728 500  B 1,00 1912,06 28,386 127  2,00 1911,31 30,072 120  3,00 1913,48 38,219 120  4,00 1914,73 21,120 125  Gesamt 1912,90 29,906 492  Gesamt 1,00 1919,26 25,384 248  2,00 1919,71 25,468 248  3,00 1920,17 30,669 248  4,00 1920,09 20,998 248  Gesamt 1919,81 25,821 992 | 1487  (*SD*=156; *N*=124)    Abhängige Variable:anker_einstein_estimate  anker_version E_Quartiles Mittelwert Std.-Abweichung N  A 1,00 1926,83 19,172 121  2,00 1927,59 16,878 128  3,00 1926,44 19,463 128  4,00 1925,54 19,492 123  Gesamt 1926,60 18,728 500  B 1,00 1912,06 28,386 127  2,00 1911,31 30,072 120  3,00 1913,48 38,219 120  4,00 1914,73 21,120 125  Gesamt 1912,90 29,906 492  Gesamt 1,00 1919,26 25,384 248  2,00 1919,71 25,468 248  3,00 1920,17 30,669 248  4,00 1920,09 20,998 248  Gesamt 1919,81 25,821 992  Deskriptive Statistiken  Abhängige Variable:anker_einstein_estimate  anker_version E_Quartiles Mittelwert Std.-Abweichung N  A 1,00 1926,83 19,172 121  2,00 1927,59 16,878 128  3,00 1926,44 19,463 128  4,00 1925,54 19,492 123  Gesamt 1926,60 18,728 500  B 1,00 1912,06 28,386 127  2,00 1911,31 30,072 120  3,00 1913,48 38,219 120  4,00 1914,73 21,120 125  Gesamt 1912,90 29,906 492  Gesamt 1,00 1919,26 25,384 248  2,00 1919,71 25,468 248  3,00 1920,17 30,669 248  4,00 1920,09 20,998 248  Gesamt 1919,81 25,821 992 |
|  | High (A) | 1585  (*SD*=124; *N*=126)    Abhängige Variable:anker_einstein_estimate  anker_version E_Quartiles Mittelwert Std.-Abweichung N  A 1,00 1926,83 19,172 121  2,00 1927,59 16,878 128  3,00 1926,44 19,463 128  4,00 1925,54 19,492 123  Gesamt 1926,60 18,728 500  B 1,00 1912,06 28,386 127  2,00 1911,31 30,072 120  3,00 1913,48 38,219 120  4,00 1914,73 21,120 125  Gesamt 1912,90 29,906 492  Gesamt 1,00 1919,26 25,384 248  2,00 1919,71 25,468 248  3,00 1920,17 30,669 248  4,00 1920,09 20,998 248  Gesamt 1919,81 25,821 992  Deskriptive Statistiken  Abhängige Variable:anker_einstein_estimate  anker_version E_Quartiles Mittelwert Std.-Abweichung N  A 1,00 1926,83 19,172 121  2,00 1927,59 16,878 128  3,00 1926,44 19,463 128  4,00 1925,54 19,492 123  Gesamt 1926,60 18,728 500  B 1,00 1912,06 28,386 127  2,00 1911,31 30,072 120  3,00 1913,48 38,219 120  4,00 1914,73 21,120 125  Gesamt 1912,90 29,906 492  Gesamt 1,00 1919,26 25,384 248  2,00 1919,71 25,468 248  3,00 1920,17 30,669 248  4,00 1920,09 20,998 248  Gesamt 1919,81 25,821 992 | 1625  (*SD*=102; *N*=124)    Abhängige Variable:anker_einstein_estimate  anker_version E_Quartiles Mittelwert Std.-Abweichung N  A 1,00 1926,83 19,172 121  2,00 1927,59 16,878 128  3,00 1926,44 19,463 128  4,00 1925,54 19,492 123  Gesamt 1926,60 18,728 500  B 1,00 1912,06 28,386 127  2,00 1911,31 30,072 120  3,00 1913,48 38,219 120  4,00 1914,73 21,120 125  Gesamt 1912,90 29,906 492  Gesamt 1,00 1919,26 25,384 248  2,00 1919,71 25,468 248  3,00 1920,17 30,669 248  4,00 1920,09 20,998 248  Gesamt 1919,81 25,821 992  Deskriptive Statistiken  Abhängige Variable:anker_einstein_estimate  anker_version E_Quartiles Mittelwert Std.-Abweichung N  A 1,00 1926,83 19,172 121  2,00 1927,59 16,878 128  3,00 1926,44 19,463 128  4,00 1925,54 19,492 123  Gesamt 1926,60 18,728 500  B 1,00 1912,06 28,386 127  2,00 1911,31 30,072 120  3,00 1913,48 38,219 120  4,00 1914,73 21,120 125  Gesamt 1912,90 29,906 492  Gesamt 1,00 1919,26 25,384 248  2,00 1919,71 25,468 248  3,00 1920,17 30,669 248  4,00 1920,09 20,998 248  Gesamt 1919,81 25,821 992 | 1624  (*SD*=128; *N*=126)    Abhängige Variable:anker_einstein_estimate  anker_version E_Quartiles Mittelwert Std.-Abweichung N  A 1,00 1926,83 19,172 121  2,00 1927,59 16,878 128  3,00 1926,44 19,463 128  4,00 1925,54 19,492 123  Gesamt 1926,60 18,728 500  B 1,00 1912,06 28,386 127  2,00 1911,31 30,072 120  3,00 1913,48 38,219 120  4,00 1914,73 21,120 125  Gesamt 1912,90 29,906 492  Gesamt 1,00 1919,26 25,384 248  2,00 1919,71 25,468 248  3,00 1920,17 30,669 248  4,00 1920,09 20,998 248  Gesamt 1919,81 25,821 992  Deskriptive Statistiken  Abhängige Variable:anker_einstein_estimate  anker_version E_Quartiles Mittelwert Std.-Abweichung N  A 1,00 1926,83 19,172 121  2,00 1927,59 16,878 128  3,00 1926,44 19,463 128  4,00 1925,54 19,492 123  Gesamt 1926,60 18,728 500  B 1,00 1912,06 28,386 127  2,00 1911,31 30,072 120  3,00 1913,48 38,219 120  4,00 1914,73 21,120 125  Gesamt 1912,90 29,906 492  Gesamt 1,00 1919,26 25,384 248  2,00 1919,71 25,468 248  3,00 1920,17 30,669 248  4,00 1920,09 20,998 248  Gesamt 1919,81 25,821 992 | 1629  (*SD*=102; *N*=124)    Abhängige Variable:anker_einstein_estimate  anker_version E_Quartiles Mittelwert Std.-Abweichung N  A 1,00 1926,83 19,172 121  2,00 1927,59 16,878 128  3,00 1926,44 19,463 128  4,00 1925,54 19,492 123  Gesamt 1926,60 18,728 500  B 1,00 1912,06 28,386 127  2,00 1911,31 30,072 120  3,00 1913,48 38,219 120  4,00 1914,73 21,120 125  Gesamt 1912,90 29,906 492  Gesamt 1,00 1919,26 25,384 248  2,00 1919,71 25,468 248  3,00 1920,17 30,669 248  4,00 1920,09 20,998 248  Gesamt 1919,81 25,821 992  Deskriptive Statistiken  Abhängige Variable:anker_einstein_estimate  anker_version E_Quartiles Mittelwert Std.-Abweichung N  A 1,00 1926,83 19,172 121  2,00 1927,59 16,878 128  3,00 1926,44 19,463 128  4,00 1925,54 19,492 123  Gesamt 1926,60 18,728 500  B 1,00 1912,06 28,386 127  2,00 1911,31 30,072 120  3,00 1913,48 38,219 120  4,00 1914,73 21,120 125  Gesamt 1912,90 29,906 492  Gesamt 1,00 1919,26 25,384 248  2,00 1919,71 25,468 248  3,00 1920,17 30,669 248  4,00 1920,09 20,998 248  Gesamt 1919,81 25,821 992 |
| Question Gandhi | Low (A) | 70  (*SD*=10; *N*=126)    Abhängige Variable:anker_einstein_estimate  anker_version E_Quartiles Mittelwert Std.-Abweichung N  A 1,00 1926,83 19,172 121  2,00 1927,59 16,878 128  3,00 1926,44 19,463 128  4,00 1925,54 19,492 123  Gesamt 1926,60 18,728 500  B 1,00 1912,06 28,386 127  2,00 1911,31 30,072 120  3,00 1913,48 38,219 120  4,00 1914,73 21,120 125  Gesamt 1912,90 29,906 492  Gesamt 1,00 1919,26 25,384 248  2,00 1919,71 25,468 248  3,00 1920,17 30,669 248  4,00 1920,09 20,998 248  Gesamt 1919,81 25,821 992  Deskriptive Statistiken  Abhängige Variable:anker_einstein_estimate  anker_version E_Quartiles Mittelwert Std.-Abweichung N  A 1,00 1926,83 19,172 121  2,00 1927,59 16,878 128  3,00 1926,44 19,463 128  4,00 1925,54 19,492 123  Gesamt 1926,60 18,728 500  B 1,00 1912,06 28,386 127  2,00 1911,31 30,072 120  3,00 1913,48 38,219 120  4,00 1914,73 21,120 125  Gesamt 1912,90 29,906 492  Gesamt 1,00 1919,26 25,384 248  2,00 1919,71 25,468 248  3,00 1920,17 30,669 248  4,00 1920,09 20,998 248  Gesamt 1919,81 25,821 992 | 70  (*SD*=10; *N*=124)    Abhängige Variable:anker_einstein_estimate  anker_version E_Quartiles Mittelwert Std.-Abweichung N  A 1,00 1926,83 19,172 121  2,00 1927,59 16,878 128  3,00 1926,44 19,463 128  4,00 1925,54 19,492 123  Gesamt 1926,60 18,728 500  B 1,00 1912,06 28,386 127  2,00 1911,31 30,072 120  3,00 1913,48 38,219 120  4,00 1914,73 21,120 125  Gesamt 1912,90 29,906 492  Gesamt 1,00 1919,26 25,384 248  2,00 1919,71 25,468 248  3,00 1920,17 30,669 248  4,00 1920,09 20,998 248  Gesamt 1919,81 25,821 992  Deskriptive Statistiken  Abhängige Variable:anker_einstein_estimate  anker_version E_Quartiles Mittelwert Std.-Abweichung N  A 1,00 1926,83 19,172 121  2,00 1927,59 16,878 128  3,00 1926,44 19,463 128  4,00 1925,54 19,492 123  Gesamt 1926,60 18,728 500  B 1,00 1912,06 28,386 127  2,00 1911,31 30,072 120  3,00 1913,48 38,219 120  4,00 1914,73 21,120 125  Gesamt 1912,90 29,906 492  Gesamt 1,00 1919,26 25,384 248  2,00 1919,71 25,468 248  3,00 1920,17 30,669 248  4,00 1920,09 20,998 248  Gesamt 1919,81 25,821 992 | 70  (*SD*=11; *N*=126)    Abhängige Variable:anker_einstein_estimate  anker_version E_Quartiles Mittelwert Std.-Abweichung N  A 1,00 1926,83 19,172 121  2,00 1927,59 16,878 128  3,00 1926,44 19,463 128  4,00 1925,54 19,492 123  Gesamt 1926,60 18,728 500  B 1,00 1912,06 28,386 127  2,00 1911,31 30,072 120  3,00 1913,48 38,219 120  4,00 1914,73 21,120 125  Gesamt 1912,90 29,906 492  Gesamt 1,00 1919,26 25,384 248  2,00 1919,71 25,468 248  3,00 1920,17 30,669 248  4,00 1920,09 20,998 248  Gesamt 1919,81 25,821 992  Deskriptive Statistiken  Abhängige Variable:anker_einstein_estimate  anker_version E_Quartiles Mittelwert Std.-Abweichung N  A 1,00 1926,83 19,172 121  2,00 1927,59 16,878 128  3,00 1926,44 19,463 128  4,00 1925,54 19,492 123  Gesamt 1926,60 18,728 500  B 1,00 1912,06 28,386 127  2,00 1911,31 30,072 120  3,00 1913,48 38,219 120  4,00 1914,73 21,120 125  Gesamt 1912,90 29,906 492  Gesamt 1,00 1919,26 25,384 248  2,00 1919,71 25,468 248  3,00 1920,17 30,669 248  4,00 1920,09 20,998 248  Gesamt 1919,81 25,821 992 | 71  (*SD*=10; *N*=124)    Abhängige Variable:anker_einstein_estimate  anker_version E_Quartiles Mittelwert Std.-Abweichung N  A 1,00 1926,83 19,172 121  2,00 1927,59 16,878 128  3,00 1926,44 19,463 128  4,00 1925,54 19,492 123  Gesamt 1926,60 18,728 500  B 1,00 1912,06 28,386 127  2,00 1911,31 30,072 120  3,00 1913,48 38,219 120  4,00 1914,73 21,120 125  Gesamt 1912,90 29,906 492  Gesamt 1,00 1919,26 25,384 248  2,00 1919,71 25,468 248  3,00 1920,17 30,669 248  4,00 1920,09 20,998 248  Gesamt 1919,81 25,821 992  Deskriptive Statistiken  Abhängige Variable:anker_einstein_estimate  anker_version E_Quartiles Mittelwert Std.-Abweichung N  A 1,00 1926,83 19,172 121  2,00 1927,59 16,878 128  3,00 1926,44 19,463 128  4,00 1925,54 19,492 123  Gesamt 1926,60 18,728 500  B 1,00 1912,06 28,386 127  2,00 1911,31 30,072 120  3,00 1913,48 38,219 120  4,00 1914,73 21,120 125  Gesamt 1912,90 29,906 492  Gesamt 1,00 1919,26 25,384 248  2,00 1919,71 25,468 248  3,00 1920,17 30,669 248  4,00 1920,09 20,998 248  Gesamt 1919,81 25,821 992 |
|  | High (B) | 78  (*SD*=9; *N*=122)    Abhängige Variable:anker_einstein_estimate  anker_version E_Quartiles Mittelwert Std.-Abweichung N  A 1,00 1926,83 19,172 121  2,00 1927,59 16,878 128  3,00 1926,44 19,463 128  4,00 1925,54 19,492 123  Gesamt 1926,60 18,728 500  B 1,00 1912,06 28,386 127  2,00 1911,31 30,072 120  3,00 1913,48 38,219 120  4,00 1914,73 21,120 125  Gesamt 1912,90 29,906 492  Gesamt 1,00 1919,26 25,384 248  2,00 1919,71 25,468 248  3,00 1920,17 30,669 248  4,00 1920,09 20,998 248  Gesamt 1919,81 25,821 992  Deskriptive Statistiken  Abhängige Variable:anker_einstein_estimate  anker_version E_Quartiles Mittelwert Std.-Abweichung N  A 1,00 1926,83 19,172 121  2,00 1927,59 16,878 128  3,00 1926,44 19,463 128  4,00 1925,54 19,492 123  Gesamt 1926,60 18,728 500  B 1,00 1912,06 28,386 127  2,00 1911,31 30,072 120  3,00 1913,48 38,219 120  4,00 1914,73 21,120 125  Gesamt 1912,90 29,906 492  Gesamt 1,00 1919,26 25,384 248  2,00 1919,71 25,468 248  3,00 1920,17 30,669 248  4,00 1920,09 20,998 248  Gesamt 1919,81 25,821 992 | 80  (*SD*=8; *N*=124)    Abhängige Variable:anker_einstein_estimate  anker_version E_Quartiles Mittelwert Std.-Abweichung N  A 1,00 1926,83 19,172 121  2,00 1927,59 16,878 128  3,00 1926,44 19,463 128  4,00 1925,54 19,492 123  Gesamt 1926,60 18,728 500  B 1,00 1912,06 28,386 127  2,00 1911,31 30,072 120  3,00 1913,48 38,219 120  4,00 1914,73 21,120 125  Gesamt 1912,90 29,906 492  Gesamt 1,00 1919,26 25,384 248  2,00 1919,71 25,468 248  3,00 1920,17 30,669 248  4,00 1920,09 20,998 248  Gesamt 1919,81 25,821 992  Deskriptive Statistiken  Abhängige Variable:anker_einstein_estimate  anker_version E_Quartiles Mittelwert Std.-Abweichung N  A 1,00 1926,83 19,172 121  2,00 1927,59 16,878 128  3,00 1926,44 19,463 128  4,00 1925,54 19,492 123  Gesamt 1926,60 18,728 500  B 1,00 1912,06 28,386 127  2,00 1911,31 30,072 120  3,00 1913,48 38,219 120  4,00 1914,73 21,120 125  Gesamt 1912,90 29,906 492  Gesamt 1,00 1919,26 25,384 248  2,00 1919,71 25,468 248  3,00 1920,17 30,669 248  4,00 1920,09 20,998 248  Gesamt 1919,81 25,821 992 | 81  (*SD*=9; *N*=122)    Abhängige Variable:anker_einstein_estimate  anker_version E_Quartiles Mittelwert Std.-Abweichung N  A 1,00 1926,83 19,172 121  2,00 1927,59 16,878 128  3,00 1926,44 19,463 128  4,00 1925,54 19,492 123  Gesamt 1926,60 18,728 500  B 1,00 1912,06 28,386 127  2,00 1911,31 30,072 120  3,00 1913,48 38,219 120  4,00 1914,73 21,120 125  Gesamt 1912,90 29,906 492  Gesamt 1,00 1919,26 25,384 248  2,00 1919,71 25,468 248  3,00 1920,17 30,669 248  4,00 1920,09 20,998 248  Gesamt 1919,81 25,821 992  Deskriptive Statistiken  Abhängige Variable:anker_einstein_estimate  anker_version E_Quartiles Mittelwert Std.-Abweichung N  A 1,00 1926,83 19,172 121  2,00 1927,59 16,878 128  3,00 1926,44 19,463 128  4,00 1925,54 19,492 123  Gesamt 1926,60 18,728 500  B 1,00 1912,06 28,386 127  2,00 1911,31 30,072 120  3,00 1913,48 38,219 120  4,00 1914,73 21,120 125  Gesamt 1912,90 29,906 492  Gesamt 1,00 1919,26 25,384 248  2,00 1919,71 25,468 248  3,00 1920,17 30,669 248  4,00 1920,09 20,998 248  Gesamt 1919,81 25,821 992 | 78  (*SD*=9; *N*=124)    Abhängige Variable:anker_einstein_estimate  anker_version E_Quartiles Mittelwert Std.-Abweichung N  A 1,00 1926,83 19,172 121  2,00 1927,59 16,878 128  3,00 1926,44 19,463 128  4,00 1925,54 19,492 123  Gesamt 1926,60 18,728 500  B 1,00 1912,06 28,386 127  2,00 1911,31 30,072 120  3,00 1913,48 38,219 120  4,00 1914,73 21,120 125  Gesamt 1912,90 29,906 492  Gesamt 1,00 1919,26 25,384 248  2,00 1919,71 25,468 248  3,00 1920,17 30,669 248  4,00 1920,09 20,998 248  Gesamt 1919,81 25,821 992  Deskriptive Statistiken  Abhängige Variable:anker_einstein_estimate  anker_version E_Quartiles Mittelwert Std.-Abweichung N  A 1,00 1926,83 19,172 121  2,00 1927,59 16,878 128  3,00 1926,44 19,463 128  4,00 1925,54 19,492 123  Gesamt 1926,60 18,728 500  B 1,00 1912,06 28,386 127  2,00 1911,31 30,072 120  3,00 1913,48 38,219 120  4,00 1914,73 21,120 125  Gesamt 1912,90 29,906 492  Gesamt 1,00 1919,26 25,384 248  2,00 1919,71 25,468 248  3,00 1920,17 30,669 248  4,00 1920,09 20,998 248  Gesamt 1919,81 25,821 992 |
| Question Sahara | Low (A) | 85  (*SD*=162; *N*=126)    Abhängige Variable:anker_einstein_estimate  anker_version E_Quartiles Mittelwert Std.-Abweichung N  A 1,00 1926,83 19,172 121  2,00 1927,59 16,878 128  3,00 1926,44 19,463 128  4,00 1925,54 19,492 123  Gesamt 1926,60 18,728 500  B 1,00 1912,06 28,386 127  2,00 1911,31 30,072 120  3,00 1913,48 38,219 120  4,00 1914,73 21,120 125  Gesamt 1912,90 29,906 492  Gesamt 1,00 1919,26 25,384 248  2,00 1919,71 25,468 248  3,00 1920,17 30,669 248  4,00 1920,09 20,998 248  Gesamt 1919,81 25,821 992  Deskriptive Statistiken  Abhängige Variable:anker_einstein_estimate  anker_version E_Quartiles Mittelwert Std.-Abweichung N  A 1,00 1926,83 19,172 121  2,00 1927,59 16,878 128  3,00 1926,44 19,463 128  4,00 1925,54 19,492 123  Gesamt 1926,60 18,728 500  B 1,00 1912,06 28,386 127  2,00 1911,31 30,072 120  3,00 1913,48 38,219 120  4,00 1914,73 21,120 125  Gesamt 1912,90 29,906 492  Gesamt 1,00 1919,26 25,384 248  2,00 1919,71 25,468 248  3,00 1920,17 30,669 248  4,00 1920,09 20,998 248  Gesamt 1919,81 25,821 992 | 83  (*SD*=124; *N*=124)    Abhängige Variable:anker_einstein_estimate  anker_version E_Quartiles Mittelwert Std.-Abweichung N  A 1,00 1926,83 19,172 121  2,00 1927,59 16,878 128  3,00 1926,44 19,463 128  4,00 1925,54 19,492 123  Gesamt 1926,60 18,728 500  B 1,00 1912,06 28,386 127  2,00 1911,31 30,072 120  3,00 1913,48 38,219 120  4,00 1914,73 21,120 125  Gesamt 1912,90 29,906 492  Gesamt 1,00 1919,26 25,384 248  2,00 1919,71 25,468 248  3,00 1920,17 30,669 248  4,00 1920,09 20,998 248  Gesamt 1919,81 25,821 992  Deskriptive Statistiken  Abhängige Variable:anker_einstein_estimate  anker_version E_Quartiles Mittelwert Std.-Abweichung N  A 1,00 1926,83 19,172 121  2,00 1927,59 16,878 128  3,00 1926,44 19,463 128  4,00 1925,54 19,492 123  Gesamt 1926,60 18,728 500  B 1,00 1912,06 28,386 127  2,00 1911,31 30,072 120  3,00 1913,48 38,219 120  4,00 1914,73 21,120 125  Gesamt 1912,90 29,906 492  Gesamt 1,00 1919,26 25,384 248  2,00 1919,71 25,468 248  3,00 1920,17 30,669 248  4,00 1920,09 20,998 248  Gesamt 1919,81 25,821 992 | 89  (*SD*=123; *N*=126)    Abhängige Variable:anker_einstein_estimate  anker_version E_Quartiles Mittelwert Std.-Abweichung N  A 1,00 1926,83 19,172 121  2,00 1927,59 16,878 128  3,00 1926,44 19,463 128  4,00 1925,54 19,492 123  Gesamt 1926,60 18,728 500  B 1,00 1912,06 28,386 127  2,00 1911,31 30,072 120  3,00 1913,48 38,219 120  4,00 1914,73 21,120 125  Gesamt 1912,90 29,906 492  Gesamt 1,00 1919,26 25,384 248  2,00 1919,71 25,468 248  3,00 1920,17 30,669 248  4,00 1920,09 20,998 248  Gesamt 1919,81 25,821 992  Deskriptive Statistiken  Abhängige Variable:anker_einstein_estimate  anker_version E_Quartiles Mittelwert Std.-Abweichung N  A 1,00 1926,83 19,172 121  2,00 1927,59 16,878 128  3,00 1926,44 19,463 128  4,00 1925,54 19,492 123  Gesamt 1926,60 18,728 500  B 1,00 1912,06 28,386 127  2,00 1911,31 30,072 120  3,00 1913,48 38,219 120  4,00 1914,73 21,120 125  Gesamt 1912,90 29,906 492  Gesamt 1,00 1919,26 25,384 248  2,00 1919,71 25,468 248  3,00 1920,17 30,669 248  4,00 1920,09 20,998 248  Gesamt 1919,81 25,821 992 | 69  (*SD*=139; *N*=124)    Abhängige Variable:anker_einstein_estimate  anker_version E_Quartiles Mittelwert Std.-Abweichung N  A 1,00 1926,83 19,172 121  2,00 1927,59 16,878 128  3,00 1926,44 19,463 128  4,00 1925,54 19,492 123  Gesamt 1926,60 18,728 500  B 1,00 1912,06 28,386 127  2,00 1911,31 30,072 120  3,00 1913,48 38,219 120  4,00 1914,73 21,120 125  Gesamt 1912,90 29,906 492  Gesamt 1,00 1919,26 25,384 248  2,00 1919,71 25,468 248  3,00 1920,17 30,669 248  4,00 1920,09 20,998 248  Gesamt 1919,81 25,821 992  Deskriptive Statistiken  Abhängige Variable:anker_einstein_estimate  anker_version E_Quartiles Mittelwert Std.-Abweichung N  A 1,00 1926,83 19,172 121  2,00 1927,59 16,878 128  3,00 1926,44 19,463 128  4,00 1925,54 19,492 123  Gesamt 1926,60 18,728 500  B 1,00 1912,06 28,386 127  2,00 1911,31 30,072 120  3,00 1913,48 38,219 120  4,00 1914,73 21,120 125  Gesamt 1912,90 29,906 492  Gesamt 1,00 1919,26 25,384 248  2,00 1919,71 25,468 248  3,00 1920,17 30,669 248  4,00 1920,09 20,998 248  Gesamt 1919,81 25,821 992 |
|  | High (B) | 113  (*SD*=123; *N*=122)    Abhängige Variable:anker_einstein_estimate  anker_version E_Quartiles Mittelwert Std.-Abweichung N  A 1,00 1926,83 19,172 121  2,00 1927,59 16,878 128  3,00 1926,44 19,463 128  4,00 1925,54 19,492 123  Gesamt 1926,60 18,728 500  B 1,00 1912,06 28,386 127  2,00 1911,31 30,072 120  3,00 1913,48 38,219 120  4,00 1914,73 21,120 125  Gesamt 1912,90 29,906 492  Gesamt 1,00 1919,26 25,384 248  2,00 1919,71 25,468 248  3,00 1920,17 30,669 248  4,00 1920,09 20,998 248  Gesamt 1919,81 25,821 992  Deskriptive Statistiken  Abhängige Variable:anker_einstein_estimate  anker_version E_Quartiles Mittelwert Std.-Abweichung N  A 1,00 1926,83 19,172 121  2,00 1927,59 16,878 128  3,00 1926,44 19,463 128  4,00 1925,54 19,492 123  Gesamt 1926,60 18,728 500  B 1,00 1912,06 28,386 127  2,00 1911,31 30,072 120  3,00 1913,48 38,219 120  4,00 1914,73 21,120 125  Gesamt 1912,90 29,906 492  Gesamt 1,00 1919,26 25,384 248  2,00 1919,71 25,468 248  3,00 1920,17 30,669 248  4,00 1920,09 20,998 248  Gesamt 1919,81 25,821 992 | 127  (*SD*=181; *N*=124)    Abhängige Variable:anker_einstein_estimate  anker_version E_Quartiles Mittelwert Std.-Abweichung N  A 1,00 1926,83 19,172 121  2,00 1927,59 16,878 128  3,00 1926,44 19,463 128  4,00 1925,54 19,492 123  Gesamt 1926,60 18,728 500  B 1,00 1912,06 28,386 127  2,00 1911,31 30,072 120  3,00 1913,48 38,219 120  4,00 1914,73 21,120 125  Gesamt 1912,90 29,906 492  Gesamt 1,00 1919,26 25,384 248  2,00 1919,71 25,468 248  3,00 1920,17 30,669 248  4,00 1920,09 20,998 248  Gesamt 1919,81 25,821 992  Deskriptive Statistiken  Abhängige Variable:anker_einstein_estimate  anker_version E_Quartiles Mittelwert Std.-Abweichung N  A 1,00 1926,83 19,172 121  2,00 1927,59 16,878 128  3,00 1926,44 19,463 128  4,00 1925,54 19,492 123  Gesamt 1926,60 18,728 500  B 1,00 1912,06 28,386 127  2,00 1911,31 30,072 120  3,00 1913,48 38,219 120  4,00 1914,73 21,120 125  Gesamt 1912,90 29,906 492  Gesamt 1,00 1919,26 25,384 248  2,00 1919,71 25,468 248  3,00 1920,17 30,669 248  4,00 1920,09 20,998 248  Gesamt 1919,81 25,821 992 | 130  (*SD*=187; *N*=122)    Abhängige Variable:anker_einstein_estimate  anker_version E_Quartiles Mittelwert Std.-Abweichung N  A 1,00 1926,83 19,172 121  2,00 1927,59 16,878 128  3,00 1926,44 19,463 128  4,00 1925,54 19,492 123  Gesamt 1926,60 18,728 500  B 1,00 1912,06 28,386 127  2,00 1911,31 30,072 120  3,00 1913,48 38,219 120  4,00 1914,73 21,120 125  Gesamt 1912,90 29,906 492  Gesamt 1,00 1919,26 25,384 248  2,00 1919,71 25,468 248  3,00 1920,17 30,669 248  4,00 1920,09 20,998 248  Gesamt 1919,81 25,821 992  Deskriptive Statistiken  Abhängige Variable:anker_einstein_estimate  anker_version E_Quartiles Mittelwert Std.-Abweichung N  A 1,00 1926,83 19,172 121  2,00 1927,59 16,878 128  3,00 1926,44 19,463 128  4,00 1925,54 19,492 123  Gesamt 1926,60 18,728 500  B 1,00 1912,06 28,386 127  2,00 1911,31 30,072 120  3,00 1913,48 38,219 120  4,00 1914,73 21,120 125  Gesamt 1912,90 29,906 492  Gesamt 1,00 1919,26 25,384 248  2,00 1919,71 25,468 248  3,00 1920,17 30,669 248  4,00 1920,09 20,998 248  Gesamt 1919,81 25,821 992 | 148  (*SD*=192; *N*=124)    Abhängige Variable:anker_einstein_estimate  anker_version E_Quartiles Mittelwert Std.-Abweichung N  A 1,00 1926,83 19,172 121  2,00 1927,59 16,878 128  3,00 1926,44 19,463 128  4,00 1925,54 19,492 123  Gesamt 1926,60 18,728 500  B 1,00 1912,06 28,386 127  2,00 1911,31 30,072 120  3,00 1913,48 38,219 120  4,00 1914,73 21,120 125  Gesamt 1912,90 29,906 492  Gesamt 1,00 1919,26 25,384 248  2,00 1919,71 25,468 248  3,00 1920,17 30,669 248  4,00 1920,09 20,998 248  Gesamt 1919,81 25,821 992  Deskriptive Statistiken  Abhängige Variable:anker_einstein_estimate  anker_version E_Quartiles Mittelwert Std.-Abweichung N  A 1,00 1926,83 19,172 121  2,00 1927,59 16,878 128  3,00 1926,44 19,463 128  4,00 1925,54 19,492 123  Gesamt 1926,60 18,728 500  B 1,00 1912,06 28,386 127  2,00 1911,31 30,072 120  3,00 1913,48 38,219 120  4,00 1914,73 21,120 125  Gesamt 1912,90 29,906 492  Gesamt 1,00 1919,26 25,384 248  2,00 1919,71 25,468 248  3,00 1920,17 30,669 248  4,00 1920,09 20,998 248  Gesamt 1919,81 25,821 992 |
| **Conscientiousness** | | **Q1 (0-25%)** | **Q2 (25-50%)** | **Q3 (50-75%)** | **Q 4 (75-100%)** |
| Question Einstein | Low (B) | 1911  (*SD*=42; *N*=127)    Abhängige Variable:anker_einstein_estimate  anker_version E_Quartiles Mittelwert Std.-Abweichung N  A 1,00 1926,83 19,172 121  2,00 1927,59 16,878 128  3,00 1926,44 19,463 128  4,00 1925,54 19,492 123  Gesamt 1926,60 18,728 500  B 1,00 1912,06 28,386 127  2,00 1911,31 30,072 120  3,00 1913,48 38,219 120  4,00 1914,73 21,120 125  Gesamt 1912,90 29,906 492  Gesamt 1,00 1919,26 25,384 248  2,00 1919,71 25,468 248  3,00 1920,17 30,669 248  4,00 1920,09 20,998 248  Gesamt 1919,81 25,821 992  Deskriptive Statistiken  Abhängige Variable:anker_einstein_estimate  anker_version E_Quartiles Mittelwert Std.-Abweichung N  A 1,00 1926,83 19,172 121  2,00 1927,59 16,878 128  3,00 1926,44 19,463 128  4,00 1925,54 19,492 123  Gesamt 1926,60 18,728 500  B 1,00 1912,06 28,386 127  2,00 1911,31 30,072 120  3,00 1913,48 38,219 120  4,00 1914,73 21,120 125  Gesamt 1912,90 29,906 492  Gesamt 1,00 1919,26 25,384 248  2,00 1919,71 25,468 248  3,00 1920,17 30,669 248  4,00 1920,09 20,998 248  Gesamt 1919,81 25,821 992 | 1917  (*SD*=27; *N*=122)    Abhängige Variable:anker_einstein_estimate  anker_version E_Quartiles Mittelwert Std.-Abweichung N  A 1,00 1926,83 19,172 121  2,00 1927,59 16,878 128  3,00 1926,44 19,463 128  4,00 1925,54 19,492 123  Gesamt 1926,60 18,728 500  B 1,00 1912,06 28,386 127  2,00 1911,31 30,072 120  3,00 1913,48 38,219 120  4,00 1914,73 21,120 125  Gesamt 1912,90 29,906 492  Gesamt 1,00 1919,26 25,384 248  2,00 1919,71 25,468 248  3,00 1920,17 30,669 248  4,00 1920,09 20,998 248  Gesamt 1919,81 25,821 992  Deskriptive Statistiken  Abhängige Variable:anker_einstein_estimate  anker_version E_Quartiles Mittelwert Std.-Abweichung N  A 1,00 1926,83 19,172 121  2,00 1927,59 16,878 128  3,00 1926,44 19,463 128  4,00 1925,54 19,492 123  Gesamt 1926,60 18,728 500  B 1,00 1912,06 28,386 127  2,00 1911,31 30,072 120  3,00 1913,48 38,219 120  4,00 1914,73 21,120 125  Gesamt 1912,90 29,906 492  Gesamt 1,00 1919,26 25,384 248  2,00 1919,71 25,468 248  3,00 1920,17 30,669 248  4,00 1920,09 20,998 248  Gesamt 1919,81 25,821 992 | 1911  (*SD*=24; *N*=115)    Abhängige Variable:anker_einstein_estimate  anker_version E_Quartiles Mittelwert Std.-Abweichung N  A 1,00 1926,83 19,172 121  2,00 1927,59 16,878 128  3,00 1926,44 19,463 128  4,00 1925,54 19,492 123  Gesamt 1926,60 18,728 500  B 1,00 1912,06 28,386 127  2,00 1911,31 30,072 120  3,00 1913,48 38,219 120  4,00 1914,73 21,120 125  Gesamt 1912,90 29,906 492  Gesamt 1,00 1919,26 25,384 248  2,00 1919,71 25,468 248  3,00 1920,17 30,669 248  4,00 1920,09 20,998 248  Gesamt 1919,81 25,821 992  Deskriptive Statistiken  Abhängige Variable:anker_einstein_estimate  anker_version E_Quartiles Mittelwert Std.-Abweichung N  A 1,00 1926,83 19,172 121  2,00 1927,59 16,878 128  3,00 1926,44 19,463 128  4,00 1925,54 19,492 123  Gesamt 1926,60 18,728 500  B 1,00 1912,06 28,386 127  2,00 1911,31 30,072 120  3,00 1913,48 38,219 120  4,00 1914,73 21,120 125  Gesamt 1912,90 29,906 492  Gesamt 1,00 1919,26 25,384 248  2,00 1919,71 25,468 248  3,00 1920,17 30,669 248  4,00 1920,09 20,998 248  Gesamt 1919,81 25,821 992 | 1913  (*SD*=22; *N*=128)    Abhängige Variable:anker_einstein_estimate  anker_version E_Quartiles Mittelwert Std.-Abweichung N  A 1,00 1926,83 19,172 121  2,00 1927,59 16,878 128  3,00 1926,44 19,463 128  4,00 1925,54 19,492 123  Gesamt 1926,60 18,728 500  B 1,00 1912,06 28,386 127  2,00 1911,31 30,072 120  3,00 1913,48 38,219 120  4,00 1914,73 21,120 125  Gesamt 1912,90 29,906 492  Gesamt 1,00 1919,26 25,384 248  2,00 1919,71 25,468 248  3,00 1920,17 30,669 248  4,00 1920,09 20,998 248  Gesamt 1919,81 25,821 992  Deskriptive Statistiken  Abhängige Variable:anker_einstein_estimate  anker_version E_Quartiles Mittelwert Std.-Abweichung N  A 1,00 1926,83 19,172 121  2,00 1927,59 16,878 128  3,00 1926,44 19,463 128  4,00 1925,54 19,492 123  Gesamt 1926,60 18,728 500  B 1,00 1912,06 28,386 127  2,00 1911,31 30,072 120  3,00 1913,48 38,219 120  4,00 1914,73 21,120 125  Gesamt 1912,90 29,906 492  Gesamt 1,00 1919,26 25,384 248  2,00 1919,71 25,468 248  3,00 1920,17 30,669 248  4,00 1920,09 20,998 248  Gesamt 1919,81 25,821 992 |
|  | High(A) | 1927  (*SD*=15; *N*=121)    Abhängige Variable:anker_einstein_estimate  anker_version E_Quartiles Mittelwert Std.-Abweichung N  A 1,00 1926,83 19,172 121  2,00 1927,59 16,878 128  3,00 1926,44 19,463 128  4,00 1925,54 19,492 123  Gesamt 1926,60 18,728 500  B 1,00 1912,06 28,386 127  2,00 1911,31 30,072 120  3,00 1913,48 38,219 120  4,00 1914,73 21,120 125  Gesamt 1912,90 29,906 492  Gesamt 1,00 1919,26 25,384 248  2,00 1919,71 25,468 248  3,00 1920,17 30,669 248  4,00 1920,09 20,998 248  Gesamt 1919,81 25,821 992  Deskriptive Statistiken  Abhängige Variable:anker_einstein_estimate  anker_version E_Quartiles Mittelwert Std.-Abweichung N  A 1,00 1926,83 19,172 121  2,00 1927,59 16,878 128  3,00 1926,44 19,463 128  4,00 1925,54 19,492 123  Gesamt 1926,60 18,728 500  B 1,00 1912,06 28,386 127  2,00 1911,31 30,072 120  3,00 1913,48 38,219 120  4,00 1914,73 21,120 125  Gesamt 1912,90 29,906 492  Gesamt 1,00 1919,26 25,384 248  2,00 1919,71 25,468 248  3,00 1920,17 30,669 248  4,00 1920,09 20,998 248  Gesamt 1919,81 25,821 992 | 1926  (*SD*=24; *N*=126)    Abhängige Variable:anker_einstein_estimate  anker_version E_Quartiles Mittelwert Std.-Abweichung N  A 1,00 1926,83 19,172 121  2,00 1927,59 16,878 128  3,00 1926,44 19,463 128  4,00 1925,54 19,492 123  Gesamt 1926,60 18,728 500  B 1,00 1912,06 28,386 127  2,00 1911,31 30,072 120  3,00 1913,48 38,219 120  4,00 1914,73 21,120 125  Gesamt 1912,90 29,906 492  Gesamt 1,00 1919,26 25,384 248  2,00 1919,71 25,468 248  3,00 1920,17 30,669 248  4,00 1920,09 20,998 248  Gesamt 1919,81 25,821 992  Deskriptive Statistiken  Abhängige Variable:anker_einstein_estimate  anker_version E_Quartiles Mittelwert Std.-Abweichung N  A 1,00 1926,83 19,172 121  2,00 1927,59 16,878 128  3,00 1926,44 19,463 128  4,00 1925,54 19,492 123  Gesamt 1926,60 18,728 500  B 1,00 1912,06 28,386 127  2,00 1911,31 30,072 120  3,00 1913,48 38,219 120  4,00 1914,73 21,120 125  Gesamt 1912,90 29,906 492  Gesamt 1,00 1919,26 25,384 248  2,00 1919,71 25,468 248  3,00 1920,17 30,669 248  4,00 1920,09 20,998 248  Gesamt 1919,81 25,821 992 | 1929  (*SD*=14; *N*=133)    Abhängige Variable:anker_einstein_estimate  anker_version E_Quartiles Mittelwert Std.-Abweichung N  A 1,00 1926,83 19,172 121  2,00 1927,59 16,878 128  3,00 1926,44 19,463 128  4,00 1925,54 19,492 123  Gesamt 1926,60 18,728 500  B 1,00 1912,06 28,386 127  2,00 1911,31 30,072 120  3,00 1913,48 38,219 120  4,00 1914,73 21,120 125  Gesamt 1912,90 29,906 492  Gesamt 1,00 1919,26 25,384 248  2,00 1919,71 25,468 248  3,00 1920,17 30,669 248  4,00 1920,09 20,998 248  Gesamt 1919,81 25,821 992  Deskriptive Statistiken  Abhängige Variable:anker_einstein_estimate  anker_version E_Quartiles Mittelwert Std.-Abweichung N  A 1,00 1926,83 19,172 121  2,00 1927,59 16,878 128  3,00 1926,44 19,463 128  4,00 1925,54 19,492 123  Gesamt 1926,60 18,728 500  B 1,00 1912,06 28,386 127  2,00 1911,31 30,072 120  3,00 1913,48 38,219 120  4,00 1914,73 21,120 125  Gesamt 1912,90 29,906 492  Gesamt 1,00 1919,26 25,384 248  2,00 1919,71 25,468 248  3,00 1920,17 30,669 248  4,00 1920,09 20,998 248  Gesamt 1919,81 25,821 992 | 1924  (*SD*=20; *N*=120)    Abhängige Variable:anker_einstein_estimate  anker_version E_Quartiles Mittelwert Std.-Abweichung N  A 1,00 1926,83 19,172 121  2,00 1927,59 16,878 128  3,00 1926,44 19,463 128  4,00 1925,54 19,492 123  Gesamt 1926,60 18,728 500  B 1,00 1912,06 28,386 127  2,00 1911,31 30,072 120  3,00 1913,48 38,219 120  4,00 1914,73 21,120 125  Gesamt 1912,90 29,906 492  Gesamt 1,00 1919,26 25,384 248  2,00 1919,71 25,468 248  3,00 1920,17 30,669 248  4,00 1920,09 20,998 248  Gesamt 1919,81 25,821 992  Deskriptive Statistiken  Abhängige Variable:anker_einstein_estimate  anker_version E_Quartiles Mittelwert Std.-Abweichung N  A 1,00 1926,83 19,172 121  2,00 1927,59 16,878 128  3,00 1926,44 19,463 128  4,00 1925,54 19,492 123  Gesamt 1926,60 18,728 500  B 1,00 1912,06 28,386 127  2,00 1911,31 30,072 120  3,00 1913,48 38,219 120  4,00 1914,73 21,120 125  Gesamt 1912,90 29,906 492  Gesamt 1,00 1919,26 25,384 248  2,00 1919,71 25,468 248  3,00 1920,17 30,669 248  4,00 1920,09 20,998 248  Gesamt 1919,81 25,821 992 |
| Question da Vinci | Low (B) | 1484  (*SD*=138; *N*=127)    Abhängige Variable:anker_einstein_estimate  anker_version E_Quartiles Mittelwert Std.-Abweichung N  A 1,00 1926,83 19,172 121  2,00 1927,59 16,878 128  3,00 1926,44 19,463 128  4,00 1925,54 19,492 123  Gesamt 1926,60 18,728 500  B 1,00 1912,06 28,386 127  2,00 1911,31 30,072 120  3,00 1913,48 38,219 120  4,00 1914,73 21,120 125  Gesamt 1912,90 29,906 492  Gesamt 1,00 1919,26 25,384 248  2,00 1919,71 25,468 248  3,00 1920,17 30,669 248  4,00 1920,09 20,998 248  Gesamt 1919,81 25,821 992  Deskriptive Statistiken  Abhängige Variable:anker_einstein_estimate  anker_version E_Quartiles Mittelwert Std.-Abweichung N  A 1,00 1926,83 19,172 121  2,00 1927,59 16,878 128  3,00 1926,44 19,463 128  4,00 1925,54 19,492 123  Gesamt 1926,60 18,728 500  B 1,00 1912,06 28,386 127  2,00 1911,31 30,072 120  3,00 1913,48 38,219 120  4,00 1914,73 21,120 125  Gesamt 1912,90 29,906 492  Gesamt 1,00 1919,26 25,384 248  2,00 1919,71 25,468 248  3,00 1920,17 30,669 248  4,00 1920,09 20,998 248  Gesamt 1919,81 25,821 992 | 1497  (*SD*=140; *N*=122)    Abhängige Variable:anker_einstein_estimate  anker_version E_Quartiles Mittelwert Std.-Abweichung N  A 1,00 1926,83 19,172 121  2,00 1927,59 16,878 128  3,00 1926,44 19,463 128  4,00 1925,54 19,492 123  Gesamt 1926,60 18,728 500  B 1,00 1912,06 28,386 127  2,00 1911,31 30,072 120  3,00 1913,48 38,219 120  4,00 1914,73 21,120 125  Gesamt 1912,90 29,906 492  Gesamt 1,00 1919,26 25,384 248  2,00 1919,71 25,468 248  3,00 1920,17 30,669 248  4,00 1920,09 20,998 248  Gesamt 1919,81 25,821 992  Deskriptive Statistiken  Abhängige Variable:anker_einstein_estimate  anker_version E_Quartiles Mittelwert Std.-Abweichung N  A 1,00 1926,83 19,172 121  2,00 1927,59 16,878 128  3,00 1926,44 19,463 128  4,00 1925,54 19,492 123  Gesamt 1926,60 18,728 500  B 1,00 1912,06 28,386 127  2,00 1911,31 30,072 120  3,00 1913,48 38,219 120  4,00 1914,73 21,120 125  Gesamt 1912,90 29,906 492  Gesamt 1,00 1919,26 25,384 248  2,00 1919,71 25,468 248  3,00 1920,17 30,669 248  4,00 1920,09 20,998 248  Gesamt 1919,81 25,821 992 | 1483  (*SD*=127; *N*=115)    Abhängige Variable:anker_einstein_estimate  anker_version E_Quartiles Mittelwert Std.-Abweichung N  A 1,00 1926,83 19,172 121  2,00 1927,59 16,878 128  3,00 1926,44 19,463 128  4,00 1925,54 19,492 123  Gesamt 1926,60 18,728 500  B 1,00 1912,06 28,386 127  2,00 1911,31 30,072 120  3,00 1913,48 38,219 120  4,00 1914,73 21,120 125  Gesamt 1912,90 29,906 492  Gesamt 1,00 1919,26 25,384 248  2,00 1919,71 25,468 248  3,00 1920,17 30,669 248  4,00 1920,09 20,998 248  Gesamt 1919,81 25,821 992  Deskriptive Statistiken  Abhängige Variable:anker_einstein_estimate  anker_version E_Quartiles Mittelwert Std.-Abweichung N  A 1,00 1926,83 19,172 121  2,00 1927,59 16,878 128  3,00 1926,44 19,463 128  4,00 1925,54 19,492 123  Gesamt 1926,60 18,728 500  B 1,00 1912,06 28,386 127  2,00 1911,31 30,072 120  3,00 1913,48 38,219 120  4,00 1914,73 21,120 125  Gesamt 1912,90 29,906 492  Gesamt 1,00 1919,26 25,384 248  2,00 1919,71 25,468 248  3,00 1920,17 30,669 248  4,00 1920,09 20,998 248  Gesamt 1919,81 25,821 992 | 1485  (*SD*=151; *N*=128)    Abhängige Variable:anker_einstein_estimate  anker_version E_Quartiles Mittelwert Std.-Abweichung N  A 1,00 1926,83 19,172 121  2,00 1927,59 16,878 128  3,00 1926,44 19,463 128  4,00 1925,54 19,492 123  Gesamt 1926,60 18,728 500  B 1,00 1912,06 28,386 127  2,00 1911,31 30,072 120  3,00 1913,48 38,219 120  4,00 1914,73 21,120 125  Gesamt 1912,90 29,906 492  Gesamt 1,00 1919,26 25,384 248  2,00 1919,71 25,468 248  3,00 1920,17 30,669 248  4,00 1920,09 20,998 248  Gesamt 1919,81 25,821 992  Deskriptive Statistiken  Abhängige Variable:anker_einstein_estimate  anker_version E_Quartiles Mittelwert Std.-Abweichung N  A 1,00 1926,83 19,172 121  2,00 1927,59 16,878 128  3,00 1926,44 19,463 128  4,00 1925,54 19,492 123  Gesamt 1926,60 18,728 500  B 1,00 1912,06 28,386 127  2,00 1911,31 30,072 120  3,00 1913,48 38,219 120  4,00 1914,73 21,120 125  Gesamt 1912,90 29,906 492  Gesamt 1,00 1919,26 25,384 248  2,00 1919,71 25,468 248  3,00 1920,17 30,669 248  4,00 1920,09 20,998 248  Gesamt 1919,81 25,821 992 |
|  | High (A) | 1616  (*SD*=111; *N*=121)    Abhängige Variable:anker_einstein_estimate  anker_version E_Quartiles Mittelwert Std.-Abweichung N  A 1,00 1926,83 19,172 121  2,00 1927,59 16,878 128  3,00 1926,44 19,463 128  4,00 1925,54 19,492 123  Gesamt 1926,60 18,728 500  B 1,00 1912,06 28,386 127  2,00 1911,31 30,072 120  3,00 1913,48 38,219 120  4,00 1914,73 21,120 125  Gesamt 1912,90 29,906 492  Gesamt 1,00 1919,26 25,384 248  2,00 1919,71 25,468 248  3,00 1920,17 30,669 248  4,00 1920,09 20,998 248  Gesamt 1919,81 25,821 992  Deskriptive Statistiken  Abhängige Variable:anker_einstein_estimate  anker_version E_Quartiles Mittelwert Std.-Abweichung N  A 1,00 1926,83 19,172 121  2,00 1927,59 16,878 128  3,00 1926,44 19,463 128  4,00 1925,54 19,492 123  Gesamt 1926,60 18,728 500  B 1,00 1912,06 28,386 127  2,00 1911,31 30,072 120  3,00 1913,48 38,219 120  4,00 1914,73 21,120 125  Gesamt 1912,90 29,906 492  Gesamt 1,00 1919,26 25,384 248  2,00 1919,71 25,468 248  3,00 1920,17 30,669 248  4,00 1920,09 20,998 248  Gesamt 1919,81 25,821 992 | 1615  (*SD*=127; *N*=126)    Abhängige Variable:anker_einstein_estimate  anker_version E_Quartiles Mittelwert Std.-Abweichung N  A 1,00 1926,83 19,172 121  2,00 1927,59 16,878 128  3,00 1926,44 19,463 128  4,00 1925,54 19,492 123  Gesamt 1926,60 18,728 500  B 1,00 1912,06 28,386 127  2,00 1911,31 30,072 120  3,00 1913,48 38,219 120  4,00 1914,73 21,120 125  Gesamt 1912,90 29,906 492  Gesamt 1,00 1919,26 25,384 248  2,00 1919,71 25,468 248  3,00 1920,17 30,669 248  4,00 1920,09 20,998 248  Gesamt 1919,81 25,821 992  Deskriptive Statistiken  Abhängige Variable:anker_einstein_estimate  anker_version E_Quartiles Mittelwert Std.-Abweichung N  A 1,00 1926,83 19,172 121  2,00 1927,59 16,878 128  3,00 1926,44 19,463 128  4,00 1925,54 19,492 123  Gesamt 1926,60 18,728 500  B 1,00 1912,06 28,386 127  2,00 1911,31 30,072 120  3,00 1913,48 38,219 120  4,00 1914,73 21,120 125  Gesamt 1912,90 29,906 492  Gesamt 1,00 1919,26 25,384 248  2,00 1919,71 25,468 248  3,00 1920,17 30,669 248  4,00 1920,09 20,998 248  Gesamt 1919,81 25,821 992 | 1620  (*SD*=109; *N*=133)    Abhängige Variable:anker_einstein_estimate  anker_version E_Quartiles Mittelwert Std.-Abweichung N  A 1,00 1926,83 19,172 121  2,00 1927,59 16,878 128  3,00 1926,44 19,463 128  4,00 1925,54 19,492 123  Gesamt 1926,60 18,728 500  B 1,00 1912,06 28,386 127  2,00 1911,31 30,072 120  3,00 1913,48 38,219 120  4,00 1914,73 21,120 125  Gesamt 1912,90 29,906 492  Gesamt 1,00 1919,26 25,384 248  2,00 1919,71 25,468 248  3,00 1920,17 30,669 248  4,00 1920,09 20,998 248  Gesamt 1919,81 25,821 992  Deskriptive Statistiken  Abhängige Variable:anker_einstein_estimate  anker_version E_Quartiles Mittelwert Std.-Abweichung N  A 1,00 1926,83 19,172 121  2,00 1927,59 16,878 128  3,00 1926,44 19,463 128  4,00 1925,54 19,492 123  Gesamt 1926,60 18,728 500  B 1,00 1912,06 28,386 127  2,00 1911,31 30,072 120  3,00 1913,48 38,219 120  4,00 1914,73 21,120 125  Gesamt 1912,90 29,906 492  Gesamt 1,00 1919,26 25,384 248  2,00 1919,71 25,468 248  3,00 1920,17 30,669 248  4,00 1920,09 20,998 248  Gesamt 1919,81 25,821 992 | 1612  (*SD*=117; *N*=120)    Abhängige Variable:anker_einstein_estimate  anker_version E_Quartiles Mittelwert Std.-Abweichung N  A 1,00 1926,83 19,172 121  2,00 1927,59 16,878 128  3,00 1926,44 19,463 128  4,00 1925,54 19,492 123  Gesamt 1926,60 18,728 500  B 1,00 1912,06 28,386 127  2,00 1911,31 30,072 120  3,00 1913,48 38,219 120  4,00 1914,73 21,120 125  Gesamt 1912,90 29,906 492  Gesamt 1,00 1919,26 25,384 248  2,00 1919,71 25,468 248  3,00 1920,17 30,669 248  4,00 1920,09 20,998 248  Gesamt 1919,81 25,821 992  Deskriptive Statistiken  Abhängige Variable:anker_einstein_estimate  anker_version E_Quartiles Mittelwert Std.-Abweichung N  A 1,00 1926,83 19,172 121  2,00 1927,59 16,878 128  3,00 1926,44 19,463 128  4,00 1925,54 19,492 123  Gesamt 1926,60 18,728 500  B 1,00 1912,06 28,386 127  2,00 1911,31 30,072 120  3,00 1913,48 38,219 120  4,00 1914,73 21,120 125  Gesamt 1912,90 29,906 492  Gesamt 1,00 1919,26 25,384 248  2,00 1919,71 25,468 248  3,00 1920,17 30,669 248  4,00 1920,09 20,998 248  Gesamt 1919,81 25,821 992 |
| Question Gandhi | Low (A) | 70  (*SD*=10; *N*=121)    Abhängige Variable:anker_einstein_estimate  anker_version E_Quartiles Mittelwert Std.-Abweichung N  A 1,00 1926,83 19,172 121  2,00 1927,59 16,878 128  3,00 1926,44 19,463 128  4,00 1925,54 19,492 123  Gesamt 1926,60 18,728 500  B 1,00 1912,06 28,386 127  2,00 1911,31 30,072 120  3,00 1913,48 38,219 120  4,00 1914,73 21,120 125  Gesamt 1912,90 29,906 492  Gesamt 1,00 1919,26 25,384 248  2,00 1919,71 25,468 248  3,00 1920,17 30,669 248  4,00 1920,09 20,998 248  Gesamt 1919,81 25,821 992  Deskriptive Statistiken  Abhängige Variable:anker_einstein_estimate  anker_version E_Quartiles Mittelwert Std.-Abweichung N  A 1,00 1926,83 19,172 121  2,00 1927,59 16,878 128  3,00 1926,44 19,463 128  4,00 1925,54 19,492 123  Gesamt 1926,60 18,728 500  B 1,00 1912,06 28,386 127  2,00 1911,31 30,072 120  3,00 1913,48 38,219 120  4,00 1914,73 21,120 125  Gesamt 1912,90 29,906 492  Gesamt 1,00 1919,26 25,384 248  2,00 1919,71 25,468 248  3,00 1920,17 30,669 248  4,00 1920,09 20,998 248  Gesamt 1919,81 25,821 992 | 69  (*SD*=11; *N*=126)    Abhängige Variable:anker_einstein_estimate  anker_version E_Quartiles Mittelwert Std.-Abweichung N  A 1,00 1926,83 19,172 121  2,00 1927,59 16,878 128  3,00 1926,44 19,463 128  4,00 1925,54 19,492 123  Gesamt 1926,60 18,728 500  B 1,00 1912,06 28,386 127  2,00 1911,31 30,072 120  3,00 1913,48 38,219 120  4,00 1914,73 21,120 125  Gesamt 1912,90 29,906 492  Gesamt 1,00 1919,26 25,384 248  2,00 1919,71 25,468 248  3,00 1920,17 30,669 248  4,00 1920,09 20,998 248  Gesamt 1919,81 25,821 992  Deskriptive Statistiken  Abhängige Variable:anker_einstein_estimate  anker_version E_Quartiles Mittelwert Std.-Abweichung N  A 1,00 1926,83 19,172 121  2,00 1927,59 16,878 128  3,00 1926,44 19,463 128  4,00 1925,54 19,492 123  Gesamt 1926,60 18,728 500  B 1,00 1912,06 28,386 127  2,00 1911,31 30,072 120  3,00 1913,48 38,219 120  4,00 1914,73 21,120 125  Gesamt 1912,90 29,906 492  Gesamt 1,00 1919,26 25,384 248  2,00 1919,71 25,468 248  3,00 1920,17 30,669 248  4,00 1920,09 20,998 248  Gesamt 1919,81 25,821 992 | 72  (*SD*=10; *N*=133)    Abhängige Variable:anker_einstein_estimate  anker_version E_Quartiles Mittelwert Std.-Abweichung N  A 1,00 1926,83 19,172 121  2,00 1927,59 16,878 128  3,00 1926,44 19,463 128  4,00 1925,54 19,492 123  Gesamt 1926,60 18,728 500  B 1,00 1912,06 28,386 127  2,00 1911,31 30,072 120  3,00 1913,48 38,219 120  4,00 1914,73 21,120 125  Gesamt 1912,90 29,906 492  Gesamt 1,00 1919,26 25,384 248  2,00 1919,71 25,468 248  3,00 1920,17 30,669 248  4,00 1920,09 20,998 248  Gesamt 1919,81 25,821 992  Deskriptive Statistiken  Abhängige Variable:anker_einstein_estimate  anker_version E_Quartiles Mittelwert Std.-Abweichung N  A 1,00 1926,83 19,172 121  2,00 1927,59 16,878 128  3,00 1926,44 19,463 128  4,00 1925,54 19,492 123  Gesamt 1926,60 18,728 500  B 1,00 1912,06 28,386 127  2,00 1911,31 30,072 120  3,00 1913,48 38,219 120  4,00 1914,73 21,120 125  Gesamt 1912,90 29,906 492  Gesamt 1,00 1919,26 25,384 248  2,00 1919,71 25,468 248  3,00 1920,17 30,669 248  4,00 1920,09 20,998 248  Gesamt 1919,81 25,821 992 | 71  (*SD*=11; *N*=120)    Abhängige Variable:anker_einstein_estimate  anker_version E_Quartiles Mittelwert Std.-Abweichung N  A 1,00 1926,83 19,172 121  2,00 1927,59 16,878 128  3,00 1926,44 19,463 128  4,00 1925,54 19,492 123  Gesamt 1926,60 18,728 500  B 1,00 1912,06 28,386 127  2,00 1911,31 30,072 120  3,00 1913,48 38,219 120  4,00 1914,73 21,120 125  Gesamt 1912,90 29,906 492  Gesamt 1,00 1919,26 25,384 248  2,00 1919,71 25,468 248  3,00 1920,17 30,669 248  4,00 1920,09 20,998 248  Gesamt 1919,81 25,821 992  Deskriptive Statistiken  Abhängige Variable:anker_einstein_estimate  anker_version E_Quartiles Mittelwert Std.-Abweichung N  A 1,00 1926,83 19,172 121  2,00 1927,59 16,878 128  3,00 1926,44 19,463 128  4,00 1925,54 19,492 123  Gesamt 1926,60 18,728 500  B 1,00 1912,06 28,386 127  2,00 1911,31 30,072 120  3,00 1913,48 38,219 120  4,00 1914,73 21,120 125  Gesamt 1912,90 29,906 492  Gesamt 1,00 1919,26 25,384 248  2,00 1919,71 25,468 248  3,00 1920,17 30,669 248  4,00 1920,09 20,998 248  Gesamt 1919,81 25,821 992 |
|  | High (B) | 79  (*SD*=8; *N*=127)    Abhängige Variable:anker_einstein_estimate  anker_version E_Quartiles Mittelwert Std.-Abweichung N  A 1,00 1926,83 19,172 121  2,00 1927,59 16,878 128  3,00 1926,44 19,463 128  4,00 1925,54 19,492 123  Gesamt 1926,60 18,728 500  B 1,00 1912,06 28,386 127  2,00 1911,31 30,072 120  3,00 1913,48 38,219 120  4,00 1914,73 21,120 125  Gesamt 1912,90 29,906 492  Gesamt 1,00 1919,26 25,384 248  2,00 1919,71 25,468 248  3,00 1920,17 30,669 248  4,00 1920,09 20,998 248  Gesamt 1919,81 25,821 992  Deskriptive Statistiken  Abhängige Variable:anker_einstein_estimate  anker_version E_Quartiles Mittelwert Std.-Abweichung N  A 1,00 1926,83 19,172 121  2,00 1927,59 16,878 128  3,00 1926,44 19,463 128  4,00 1925,54 19,492 123  Gesamt 1926,60 18,728 500  B 1,00 1912,06 28,386 127  2,00 1911,31 30,072 120  3,00 1913,48 38,219 120  4,00 1914,73 21,120 125  Gesamt 1912,90 29,906 492  Gesamt 1,00 1919,26 25,384 248  2,00 1919,71 25,468 248  3,00 1920,17 30,669 248  4,00 1920,09 20,998 248  Gesamt 1919,81 25,821 992 | 79  (*SD*=8; *N*=122)    Abhängige Variable:anker_einstein_estimate  anker_version E_Quartiles Mittelwert Std.-Abweichung N  A 1,00 1926,83 19,172 121  2,00 1927,59 16,878 128  3,00 1926,44 19,463 128  4,00 1925,54 19,492 123  Gesamt 1926,60 18,728 500  B 1,00 1912,06 28,386 127  2,00 1911,31 30,072 120  3,00 1913,48 38,219 120  4,00 1914,73 21,120 125  Gesamt 1912,90 29,906 492  Gesamt 1,00 1919,26 25,384 248  2,00 1919,71 25,468 248  3,00 1920,17 30,669 248  4,00 1920,09 20,998 248  Gesamt 1919,81 25,821 992  Deskriptive Statistiken  Abhängige Variable:anker_einstein_estimate  anker_version E_Quartiles Mittelwert Std.-Abweichung N  A 1,00 1926,83 19,172 121  2,00 1927,59 16,878 128  3,00 1926,44 19,463 128  4,00 1925,54 19,492 123  Gesamt 1926,60 18,728 500  B 1,00 1912,06 28,386 127  2,00 1911,31 30,072 120  3,00 1913,48 38,219 120  4,00 1914,73 21,120 125  Gesamt 1912,90 29,906 492  Gesamt 1,00 1919,26 25,384 248  2,00 1919,71 25,468 248  3,00 1920,17 30,669 248  4,00 1920,09 20,998 248  Gesamt 1919,81 25,821 992 | 80  (*SD*=9; *N*=115)    Abhängige Variable:anker_einstein_estimate  anker_version E_Quartiles Mittelwert Std.-Abweichung N  A 1,00 1926,83 19,172 121  2,00 1927,59 16,878 128  3,00 1926,44 19,463 128  4,00 1925,54 19,492 123  Gesamt 1926,60 18,728 500  B 1,00 1912,06 28,386 127  2,00 1911,31 30,072 120  3,00 1913,48 38,219 120  4,00 1914,73 21,120 125  Gesamt 1912,90 29,906 492  Gesamt 1,00 1919,26 25,384 248  2,00 1919,71 25,468 248  3,00 1920,17 30,669 248  4,00 1920,09 20,998 248  Gesamt 1919,81 25,821 992  Deskriptive Statistiken  Abhängige Variable:anker_einstein_estimate  anker_version E_Quartiles Mittelwert Std.-Abweichung N  A 1,00 1926,83 19,172 121  2,00 1927,59 16,878 128  3,00 1926,44 19,463 128  4,00 1925,54 19,492 123  Gesamt 1926,60 18,728 500  B 1,00 1912,06 28,386 127  2,00 1911,31 30,072 120  3,00 1913,48 38,219 120  4,00 1914,73 21,120 125  Gesamt 1912,90 29,906 492  Gesamt 1,00 1919,26 25,384 248  2,00 1919,71 25,468 248  3,00 1920,17 30,669 248  4,00 1920,09 20,998 248  Gesamt 1919,81 25,821 992 | 77  (*SD*=10; *N*=128)    Abhängige Variable:anker_einstein_estimate  anker_version E_Quartiles Mittelwert Std.-Abweichung N  A 1,00 1926,83 19,172 121  2,00 1927,59 16,878 128  3,00 1926,44 19,463 128  4,00 1925,54 19,492 123  Gesamt 1926,60 18,728 500  B 1,00 1912,06 28,386 127  2,00 1911,31 30,072 120  3,00 1913,48 38,219 120  4,00 1914,73 21,120 125  Gesamt 1912,90 29,906 492  Gesamt 1,00 1919,26 25,384 248  2,00 1919,71 25,468 248  3,00 1920,17 30,669 248  4,00 1920,09 20,998 248  Gesamt 1919,81 25,821 992  Deskriptive Statistiken  Abhängige Variable:anker_einstein_estimate  anker_version E_Quartiles Mittelwert Std.-Abweichung N  A 1,00 1926,83 19,172 121  2,00 1927,59 16,878 128  3,00 1926,44 19,463 128  4,00 1925,54 19,492 123  Gesamt 1926,60 18,728 500  B 1,00 1912,06 28,386 127  2,00 1911,31 30,072 120  3,00 1913,48 38,219 120  4,00 1914,73 21,120 125  Gesamt 1912,90 29,906 492  Gesamt 1,00 1919,26 25,384 248  2,00 1919,71 25,468 248  3,00 1920,17 30,669 248  4,00 1920,09 20,998 248  Gesamt 1919,81 25,821 992 |
| Question Sahara | Low (A) | 103  (*SD*=169; *N*=121)    Abhängige Variable:anker_einstein_estimate  anker_version E_Quartiles Mittelwert Std.-Abweichung N  A 1,00 1926,83 19,172 121  2,00 1927,59 16,878 128  3,00 1926,44 19,463 128  4,00 1925,54 19,492 123  Gesamt 1926,60 18,728 500  B 1,00 1912,06 28,386 127  2,00 1911,31 30,072 120  3,00 1913,48 38,219 120  4,00 1914,73 21,120 125  Gesamt 1912,90 29,906 492  Gesamt 1,00 1919,26 25,384 248  2,00 1919,71 25,468 248  3,00 1920,17 30,669 248  4,00 1920,09 20,998 248  Gesamt 1919,81 25,821 992  Deskriptive Statistiken  Abhängige Variable:anker_einstein_estimate  anker_version E_Quartiles Mittelwert Std.-Abweichung N  A 1,00 1926,83 19,172 121  2,00 1927,59 16,878 128  3,00 1926,44 19,463 128  4,00 1925,54 19,492 123  Gesamt 1926,60 18,728 500  B 1,00 1912,06 28,386 127  2,00 1911,31 30,072 120  3,00 1913,48 38,219 120  4,00 1914,73 21,120 125  Gesamt 1912,90 29,906 492  Gesamt 1,00 1919,26 25,384 248  2,00 1919,71 25,468 248  3,00 1920,17 30,669 248  4,00 1920,09 20,998 248  Gesamt 1919,81 25,821 992 | 69  (*SD*=90; *N*=126)    Abhängige Variable:anker_einstein_estimate  anker_version E_Quartiles Mittelwert Std.-Abweichung N  A 1,00 1926,83 19,172 121  2,00 1927,59 16,878 128  3,00 1926,44 19,463 128  4,00 1925,54 19,492 123  Gesamt 1926,60 18,728 500  B 1,00 1912,06 28,386 127  2,00 1911,31 30,072 120  3,00 1913,48 38,219 120  4,00 1914,73 21,120 125  Gesamt 1912,90 29,906 492  Gesamt 1,00 1919,26 25,384 248  2,00 1919,71 25,468 248  3,00 1920,17 30,669 248  4,00 1920,09 20,998 248  Gesamt 1919,81 25,821 992  Deskriptive Statistiken  Abhängige Variable:anker_einstein_estimate  anker_version E_Quartiles Mittelwert Std.-Abweichung N  A 1,00 1926,83 19,172 121  2,00 1927,59 16,878 128  3,00 1926,44 19,463 128  4,00 1925,54 19,492 123  Gesamt 1926,60 18,728 500  B 1,00 1912,06 28,386 127  2,00 1911,31 30,072 120  3,00 1913,48 38,219 120  4,00 1914,73 21,120 125  Gesamt 1912,90 29,906 492  Gesamt 1,00 1919,26 25,384 248  2,00 1919,71 25,468 248  3,00 1920,17 30,669 248  4,00 1920,09 20,998 248  Gesamt 1919,81 25,821 992 | 78  (*SD*=140; *N*=133)    Abhängige Variable:anker_einstein_estimate  anker_version E_Quartiles Mittelwert Std.-Abweichung N  A 1,00 1926,83 19,172 121  2,00 1927,59 16,878 128  3,00 1926,44 19,463 128  4,00 1925,54 19,492 123  Gesamt 1926,60 18,728 500  B 1,00 1912,06 28,386 127  2,00 1911,31 30,072 120  3,00 1913,48 38,219 120  4,00 1914,73 21,120 125  Gesamt 1912,90 29,906 492  Gesamt 1,00 1919,26 25,384 248  2,00 1919,71 25,468 248  3,00 1920,17 30,669 248  4,00 1920,09 20,998 248  Gesamt 1919,81 25,821 992  Deskriptive Statistiken  Abhängige Variable:anker_einstein_estimate  anker_version E_Quartiles Mittelwert Std.-Abweichung N  A 1,00 1926,83 19,172 121  2,00 1927,59 16,878 128  3,00 1926,44 19,463 128  4,00 1925,54 19,492 123  Gesamt 1926,60 18,728 500  B 1,00 1912,06 28,386 127  2,00 1911,31 30,072 120  3,00 1913,48 38,219 120  4,00 1914,73 21,120 125  Gesamt 1912,90 29,906 492  Gesamt 1,00 1919,26 25,384 248  2,00 1919,71 25,468 248  3,00 1920,17 30,669 248  4,00 1920,09 20,998 248  Gesamt 1919,81 25,821 992 | 77  (*SD*=140; *N*=120)    Abhängige Variable:anker_einstein_estimate  anker_version E_Quartiles Mittelwert Std.-Abweichung N  A 1,00 1926,83 19,172 121  2,00 1927,59 16,878 128  3,00 1926,44 19,463 128  4,00 1925,54 19,492 123  Gesamt 1926,60 18,728 500  B 1,00 1912,06 28,386 127  2,00 1911,31 30,072 120  3,00 1913,48 38,219 120  4,00 1914,73 21,120 125  Gesamt 1912,90 29,906 492  Gesamt 1,00 1919,26 25,384 248  2,00 1919,71 25,468 248  3,00 1920,17 30,669 248  4,00 1920,09 20,998 248  Gesamt 1919,81 25,821 992  Deskriptive Statistiken  Abhängige Variable:anker_einstein_estimate  anker_version E_Quartiles Mittelwert Std.-Abweichung N  A 1,00 1926,83 19,172 121  2,00 1927,59 16,878 128  3,00 1926,44 19,463 128  4,00 1925,54 19,492 123  Gesamt 1926,60 18,728 500  B 1,00 1912,06 28,386 127  2,00 1911,31 30,072 120  3,00 1913,48 38,219 120  4,00 1914,73 21,120 125  Gesamt 1912,90 29,906 492  Gesamt 1,00 1919,26 25,384 248  2,00 1919,71 25,468 248  3,00 1920,17 30,669 248  4,00 1920,09 20,998 248  Gesamt 1919,81 25,821 992 |
|  | High (B) | 126  (*SD*=171; *N*=127)    Abhängige Variable:anker_einstein_estimate  anker_version E_Quartiles Mittelwert Std.-Abweichung N  A 1,00 1926,83 19,172 121  2,00 1927,59 16,878 128  3,00 1926,44 19,463 128  4,00 1925,54 19,492 123  Gesamt 1926,60 18,728 500  B 1,00 1912,06 28,386 127  2,00 1911,31 30,072 120  3,00 1913,48 38,219 120  4,00 1914,73 21,120 125  Gesamt 1912,90 29,906 492  Gesamt 1,00 1919,26 25,384 248  2,00 1919,71 25,468 248  3,00 1920,17 30,669 248  4,00 1920,09 20,998 248  Gesamt 1919,81 25,821 992  Deskriptive Statistiken  Abhängige Variable:anker_einstein_estimate  anker_version E_Quartiles Mittelwert Std.-Abweichung N  A 1,00 1926,83 19,172 121  2,00 1927,59 16,878 128  3,00 1926,44 19,463 128  4,00 1925,54 19,492 123  Gesamt 1926,60 18,728 500  B 1,00 1912,06 28,386 127  2,00 1911,31 30,072 120  3,00 1913,48 38,219 120  4,00 1914,73 21,120 125  Gesamt 1912,90 29,906 492  Gesamt 1,00 1919,26 25,384 248  2,00 1919,71 25,468 248  3,00 1920,17 30,669 248  4,00 1920,09 20,998 248  Gesamt 1919,81 25,821 992 | 117  (*SD*=151; *N*=122)    Abhängige Variable:anker_einstein_estimate  anker_version E_Quartiles Mittelwert Std.-Abweichung N  A 1,00 1926,83 19,172 121  2,00 1927,59 16,878 128  3,00 1926,44 19,463 128  4,00 1925,54 19,492 123  Gesamt 1926,60 18,728 500  B 1,00 1912,06 28,386 127  2,00 1911,31 30,072 120  3,00 1913,48 38,219 120  4,00 1914,73 21,120 125  Gesamt 1912,90 29,906 492  Gesamt 1,00 1919,26 25,384 248  2,00 1919,71 25,468 248  3,00 1920,17 30,669 248  4,00 1920,09 20,998 248  Gesamt 1919,81 25,821 992  Deskriptive Statistiken  Abhängige Variable:anker_einstein_estimate  anker_version E_Quartiles Mittelwert Std.-Abweichung N  A 1,00 1926,83 19,172 121  2,00 1927,59 16,878 128  3,00 1926,44 19,463 128  4,00 1925,54 19,492 123  Gesamt 1926,60 18,728 500  B 1,00 1912,06 28,386 127  2,00 1911,31 30,072 120  3,00 1913,48 38,219 120  4,00 1914,73 21,120 125  Gesamt 1912,90 29,906 492  Gesamt 1,00 1919,26 25,384 248  2,00 1919,71 25,468 248  3,00 1920,17 30,669 248  4,00 1920,09 20,998 248  Gesamt 1919,81 25,821 992 | 128  (*SD*=152; *N*=115)    Abhängige Variable:anker_einstein_estimate  anker_version E_Quartiles Mittelwert Std.-Abweichung N  A 1,00 1926,83 19,172 121  2,00 1927,59 16,878 128  3,00 1926,44 19,463 128  4,00 1925,54 19,492 123  Gesamt 1926,60 18,728 500  B 1,00 1912,06 28,386 127  2,00 1911,31 30,072 120  3,00 1913,48 38,219 120  4,00 1914,73 21,120 125  Gesamt 1912,90 29,906 492  Gesamt 1,00 1919,26 25,384 248  2,00 1919,71 25,468 248  3,00 1920,17 30,669 248  4,00 1920,09 20,998 248  Gesamt 1919,81 25,821 992  Deskriptive Statistiken  Abhängige Variable:anker_einstein_estimate  anker_version E_Quartiles Mittelwert Std.-Abweichung N  A 1,00 1926,83 19,172 121  2,00 1927,59 16,878 128  3,00 1926,44 19,463 128  4,00 1925,54 19,492 123  Gesamt 1926,60 18,728 500  B 1,00 1912,06 28,386 127  2,00 1911,31 30,072 120  3,00 1913,48 38,219 120  4,00 1914,73 21,120 125  Gesamt 1912,90 29,906 492  Gesamt 1,00 1919,26 25,384 248  2,00 1919,71 25,468 248  3,00 1920,17 30,669 248  4,00 1920,09 20,998 248  Gesamt 1919,81 25,821 992 | 146  (*SD*=210; *N*=128)    Abhängige Variable:anker_einstein_estimate  anker_version E_Quartiles Mittelwert Std.-Abweichung N  A 1,00 1926,83 19,172 121  2,00 1927,59 16,878 128  3,00 1926,44 19,463 128  4,00 1925,54 19,492 123  Gesamt 1926,60 18,728 500  B 1,00 1912,06 28,386 127  2,00 1911,31 30,072 120  3,00 1913,48 38,219 120  4,00 1914,73 21,120 125  Gesamt 1912,90 29,906 492  Gesamt 1,00 1919,26 25,384 248  2,00 1919,71 25,468 248  3,00 1920,17 30,669 248  4,00 1920,09 20,998 248  Gesamt 1919,81 25,821 992  Deskriptive Statistiken  Abhängige Variable:anker_einstein_estimate  anker_version E_Quartiles Mittelwert Std.-Abweichung N  A 1,00 1926,83 19,172 121  2,00 1927,59 16,878 128  3,00 1926,44 19,463 128  4,00 1925,54 19,492 123  Gesamt 1926,60 18,728 500  B 1,00 1912,06 28,386 127  2,00 1911,31 30,072 120  3,00 1913,48 38,219 120  4,00 1914,73 21,120 125  Gesamt 1912,90 29,906 492  Gesamt 1,00 1919,26 25,384 248  2,00 1919,71 25,468 248  3,00 1920,17 30,669 248  4,00 1920,09 20,998 248  Gesamt 1919,81 25,821 992 |

Note. Q1-Q4: Quartiles for each personality factor for participants with the lowest (Q1) to highest (Q4) personality scores. In each cell, the means of the estimates within the given quartile of participants are depicted. (A) and (B): The question set existed in two versions (A and B), each with two high and with two low anchored questions (in reversed order).

| **Supplementary Table S2. Bayesian ANOVAs for interactions between personality and anchors** | | | | | | | | | | | |
| --- | --- | --- | --- | --- | --- | --- | --- | --- | --- | --- | --- |
| **Neuroticism** | | **P(M)** | | **P(M\|data)** | | **BF _M_** | | **BF _01_** | | **error %** | |
| Null model (incl main effects)  N quartiles ✻  *anchor Einstein* |  | 0.500 |  | 0.726 |  | 2.645 |  | 1.000 |  |  |  |
|  |  | 0.500 |  | 0.274 |  | 0.378 |  | **2.645** |  | 2.298 |  |
|  |  |  |  |  |  |  |  |  |  |  |  |
| Null model (incl main effects)  Nquartiles ✻  *anchor Davinci* |  | 0.500 |  | 0.964 |  | 27.121 |  | 1.000 |  |  |  |
|  |  | 0.500 |  | 0.036 |  | 0.037 |  | **27.121** |  | 12.79 |  |
|  | | | | | | | | | | | |
| Null model (incl main effects)  Nquartiles ✻  *anchorGhandi* |  | 0.500 |  | 0.987 |  | 73.678 |  | 1.000 |  |  |  |
|  |  | 0.500 |  | 0.013 |  | 0.014 |  | **73.678** |  | 4.98 |  |
|  | | | | | | | | | | | |
| Null model (incl main effects)  N quartiles ✻  *anchor Sahara* |  | 0.500 |  | 0.984 |  | 62.731 |  | 1.000 |  |  |  |
|  |  | 0.500 |  | 0.016 |  | 0.016 |  | **62.731** |  | 8.69 |  |
| **Extraversion** | |  | |  | |  | |  | |  | |
| Null model (incl main effects)  E quartiles ✻  *anchor Einstein* |  | 0.500 |  | 0.984 |  | 63.205 |  | 1.000 |  |  |  |
|  |  | 0.500 |  | 0.016 |  | 0.016 |  | **63.205** |  | 7.32 |  |
|  | | | | | | | | | | | |
| Null model (incl main effects)  E quartiles ✻ *anchor Davinci* |  | 0.500 |  | 0.981 |  | 50.556 |  | 1.000 |  |  |  |
|  |  | 0.500 |  | 0.019 |  | 0.020 |  | **50.556** |  | 2.86 |  |
|  | | | | | | | | | | | |
| Null model (incl main effects)  E quartiles ✻ *anchor Ghandi* |  | 0.500 |  | 0.524 |  | 1.103 |  | 1.000 |  |  |  |
|  |  | 0.500 |  | 0.476 |  | 0.907 |  | **1.103** |  | 9.66 |  |
|  | | | | | | | | | | | |
| Null model (incl main effects)  E quartiles ✻ *anchor Sahara* |  | 0.500 |  | 0.903 |  | 9.281 |  | 1.000 |  |  |  |
|  |  | 0.500 |  | 0.097 |  | 0.108 |  | **9.281** |  | 2.99 |  |
| **Openness** | |  | |  | |  | |  | |  | |
| Null model (incl main effects)  O quartiles ✻*anchor Einstein* |  | 0.500 |  | 0.977 |  | 42.792 |  | 1.000 |  |  |  |
|  |  | 0.500 |  | 0.023 |  | 0.023 |  | **42.792** |  | 10.22 |  |
|  | | | | | | | | | | | |
| Null model (incl main effects)  O quartiles ✻*anchor Davinci* |  | 0.500 |  | 0.981 |  | 50.556 |  | 1.000 |  |  |  |
|  |  | 0.500 |  | 0.019 |  | 0.020 |  | **50.556** |  | 2.86 |  |
|  | | | | | | | | | | | |
| Null model (incl main effects)  O quartiles ✻ a*nchor Ghandi* |  | 0.500 |  | 0.984 |  | 60.487 |  | 1.000 |  |  |  |
|  |  | 0.500 |  | 0.016 |  | 0.017 |  | **60.487** |  | 13.51 |  |
|  | | | | | | | | | | | |
| Null model (incl main effects)  O quartiles ✻ *anchor Sahara* |  | 0.500 |  | 0.633 |  | 1.726 |  | 1.000 |  |  |  |
|  |  | 0.500 |  | 0.367 |  | 0.579 |  | **1.726** |  | 1.74 |  |
| **Agreeableness** | |  | |  | |  | |  | |  | |
| Null model (incl main effects)  A quartiles ✻*anchor Einstein* |  | 0.500 |  | 0.866 |  | 6.483 |  | 1.000 |  |  |  |
|  |  | 0.500 |  | 0.134 |  | 0.154 |  | **6.483** |  | 2.18 |  |
|  | | | | | | | | | | | |
| Null model (incl main effects)  A quartiles ✻*anchor Davinci* |  | 0.500 |  | 0.843 |  | 5.361 |  | 1.000 |  |  |  |
|  |  | 0.500 |  | 0.157 |  | 0.187 |  | **5.361** |  | 2.14 |  |
|  | | | | | | | | | | | |
| Null model (incl main effects)  A quartiles ✻*anchor Ghandi* |  | 0.500 |  | 0.905 |  | 9.530 |  | 1.000 |  |  |  |
|  |  | 0.500 |  | 0.095 |  | 0.105 |  | **9.530** |  | 2.67 |  |
|  | | | | | | | | | | | |
| Null model (incl main effects)  A quartiles ✻*anchor Sahara* |  | 0.500 |  | 0.962 |  | 25.585 |  | 1.000 |  |  |  |
|  |  | 0.500 |  | 0.038 |  | 0.039 |  | **25.585** |  | 2.59 |  |
| **Conscientiousness** | |  | |  | |  | |  | |  | |
| Null model (incl main effects)  Cquartiles ✻*anchor Einstein* |  | 0.500 |  | 0.944 |  | 16.825 |  | 1.000 |  |  |  |
|  |  | 0.500 |  | 0.056 |  | 0.059 |  | **16.825** |  | 2.70 |  |
|  | | | | | | | | | | | |
| Null model (incl main effects)  C quartiles ✻*anchor Davinci* |  | 0.500 |  | 0.991 |  | 111.963 |  | 1.000 |  |  |  |
|  |  | 0.500 |  | 0.009 |  | 0.009 |  | **111.963** |  | 2.21 |  |
|  | | | | | | | | | | | |
| Null model (incl main effects)  C quartiles ✻*anchor Ghandi* |  | 0.500 |  | 0.896 |  | 8.653 |  | 1.000 |  |  |  |
|  |  | 0.500 |  | 0.104 |  | 0.116 |  | **8.653** |  | 3.83 |  |
|  | | | | | | | | | | | |
| Null model (incl main effects)  C quartiles ✻*anchor Sahara* |  | 0.500 |  | 0.975 |  | 38.341 |  | 1.000 |  |  |  |
|  |  | 0.500 |  | 0.025 |  | 0.026 |  | **38.341** |  | 3.53 |  |

Note. All models are compared to the best model, including the main effects. BF₀₁ displays how many times more likely the non-existence of interaction is. N = Neuroticism, E = Extraversion, O = Openness, A = Agreeableness, C = Conscientiousness.
